# Supplementary material for: Unifying package managers, workflow engines, and containers: Computational reproducibility with BioNix
Source: Gigascience. 2020 Nov 18;9(11):giaa121. doi: 10.1093/gigascience/giaa121 (PMC7672450; doi:10.1093/gigascience/giaa121)
Supplement: giaa121_GIGA-D-19-00324_Original_Submission [file giaa121_giga-d-19-00324_original_submission.pdf]

GigaScience
Unifying package managers, workflow engines, and containers with BioNix

for

computational reproducibility

--Manuscript Draft--

**Manuscript Number:**	GIGA-D-19-00324
**Full Title:**	Unifying package managers, workflow engines, and containers with BioNix<br>for<br>computational reproducibility
**Article Type:**	Technical Note
**Funding Information:**	Australian National Health and Medical Research Council (1054618) — Dr Anthony T. Papenfuss<br>Australian National Health and Medical Research Council (1116955) — Dr Anthony T. Papenfuss
**Abstract:**	Motivation: A challenge for computational biologists is to make our analyses reproducible -- that is, easy to rerun, combine, and share, with the assurance that equivalent runs will generate identical results. Current best practice aims at this using a combination of package managers, workflow engines, and containers.<br><br>Results: We present BioNix, a lightweight library built on the Nix deployment system. BioNix manages software dependencies, computational environments, and workflow stages together using a single abstraction: pure functions. This lets us specify workflows in a way that is more reproducible and modular than current best practices.<br><br>Availability and implementation: BioNix is implemented in the Nix expression language and is released on GitHub under the 3-clause BSD license: https://github.com/PapenfussLab/bionix.
**Corresponding Author:**	Justin Bedo<br><br>AUSTRALIA
**Corresponding Author Secondary Information:**	
**Corresponding Author's Institution:**	
**Corresponding Author's Secondary Institution:**	
**First Author:**	Justin Bedo
**First Author Secondary Information:**	
**Order of Authors:**	Justin Bedo<br>Leon Di Stefano<br>Anthony T. Papenfuss
**Order of Authors Secondary Information:**	
**Additional Information:**	

Powered by Editorial Manager® and ProduXion Manager® from Aries Systems Corporation

Question	Response
Are you submitting this manuscript to a special series or article collection?	No
**Experimental design and statistics**<br><br>Full details of the experimental design and statistical methods used should be given in the Methods section, as detailed in our Minimum Standards Reporting Checklist. Information essential to interpreting the data presented should be made available in the figure legends.<br><br>Have you included all the information requested in your manuscript?	Yes
**Resources**<br><br>A description of all resources used, including antibodies, cell lines, animals and software tools, with enough information to allow them to be uniquely identified, should be included in the Methods section. Authors are strongly encouraged to cite Research Resource Identifiers (RRIDs) for antibodies, model organisms and tools, where possible.<br><br>Have you included the information requested as detailed in our Minimum Standards Reporting Checklist?	Yes
**Availability of data and materials**<br><br>All datasets and code on which the conclusions of the paper rely must be either included in your submission or deposited in publicly available repositories (where available and ethically appropriate), referencing such data using a unique identifier in the references and in the "Availability of Data and Materials" section of your manuscript.	Yes

Have you have met the above requirement as detailed in our Minimum Standards Reporting Checklist?	

Powered by Editorial Manager® and ProduXion Manager® from Aries Systems Corporation

Manuscript

Click here to download Manuscript paper.pdf

Placeholder for OUP logo oup.pdf

GigaScience, 2017, 1–8

doi: xx.xxxx/xxxx
Manuscript in Preparation
Technical Note

TECHNICAL NOTE

Unifying package managers, workflow engines, and containers with BioNix for computational reproducibility

Justin Bedő[1,2,3,*], Leon Di Stefano[1] and Anthony T. Papenfuss[1,2,4,5,6]

[1]Bioinformatics Division, Walter and Eliza Hall Institute of Medical Research, Parkville, 3052, Victoria, Australia and [2]Peter MacCallum Cancer Centre, Melbourne, VIC 3000, Australia and [3]Department of Computing and Information Systems, University of Melbourne, Melbourne, VIC 3010, Australia and [4]Department of Medical Biology, University of Melbourne, Melbourne, VIC 3010, Australia and [5]Sir Peter MacCallum Department of Oncology, University of Melbourne, Melbourne, VIC 3010, Australia and [6]School of Mathematics and Statistics, University of Melbourne, Melbourne, VIC 3010, Australia

[*] Correspondence author: Justin Bedő, Bioinformatics Division, Walter and Eliza Hall Institute of Medical Research, 3010. Email: bedo.j@wehi.edu.au

Abstract

Motivation: A challenge for computational biologists is to make our analyses reproducible – that is, easy to rerun, combine, and share, with the assurance that equivalent runs will generate identical results. Current best practice aims at this using a combination of package managers, workflow engines, and containers.
Results: We present *BioNix*, a lightweight library built on the Nix deployment system. BioNix manages software dependencies, computational environments, and workflow stages together using a single abstraction: pure functions. This lets us specify workflows in a way that is more reproducible and modular than current best practices.
Availability and implementation: BioNix is implemented in the Nix expression language and is released on GitHub under the 3-clause BSD license:
https://github.com/PapenfussLab/bionix.

Introduction

There are many aspects to the ongoing reproducibility crisis in science – imprecise laboratory protocols, selective reporting, poor use of statistical methods [1, 2] – but for researchers in bioinformatics the most important of these is *computational reproducibility*. Three main challenges exist in practice:

i. *Managing software versions and dependencies*. This is commonly handled with *package managers* (e.g., Conda [3]), which provide both a central repository of software and tools to manage installation on a user's system.
ii. *Managing computational environments*. This is commonly handled with *containers* (e.g., Docker [4], Singularity [5]) or *virtual machines*; these provide controlled environments within which workflows can be executed.
iii. *Managing workflows*. This is commonly handled with *workflow engines* (e.g., Toil [6], Ruffus [7]), which manage *stages*[1] and their execution, providing features like parallelism, remote building, resumability, and logging.

The combination of technologies to address these issues are referred to as a *reproducibility stack* by Grüning, Chilton, Köster, et al. [8].

Our contributions. We present BioNix, a lightweight library that cleanly overcomes all three of these challenges within the one system, resulting in simpler bioinformatics workflow specification and improving reproducibility.

Two distinctive aspects of BioNix's design enable these improvements. The first is that BioNix is built on *Nix*, a next generation cross-platform software deployment system. The second is that in BioNix, stages of a workflow are modelled as *pure functions* – i.e., functions that are free of side effects.

These design choices give BioNix several novel features, which we explain using the complete workflow and associated build graph depicted in Example 1:

i. BioNix *manages both software and workflows within the one system*. The build graph in Example 1 has nodes corresponding not just to workflow stages and inputs, but also to software dependencies.
ii. Each stage of a BioNix workflow *implicitly specifies its entire*

1 We define a *stage* as the concrete execution of one or more executables on one or more input files, producing one or more output files.

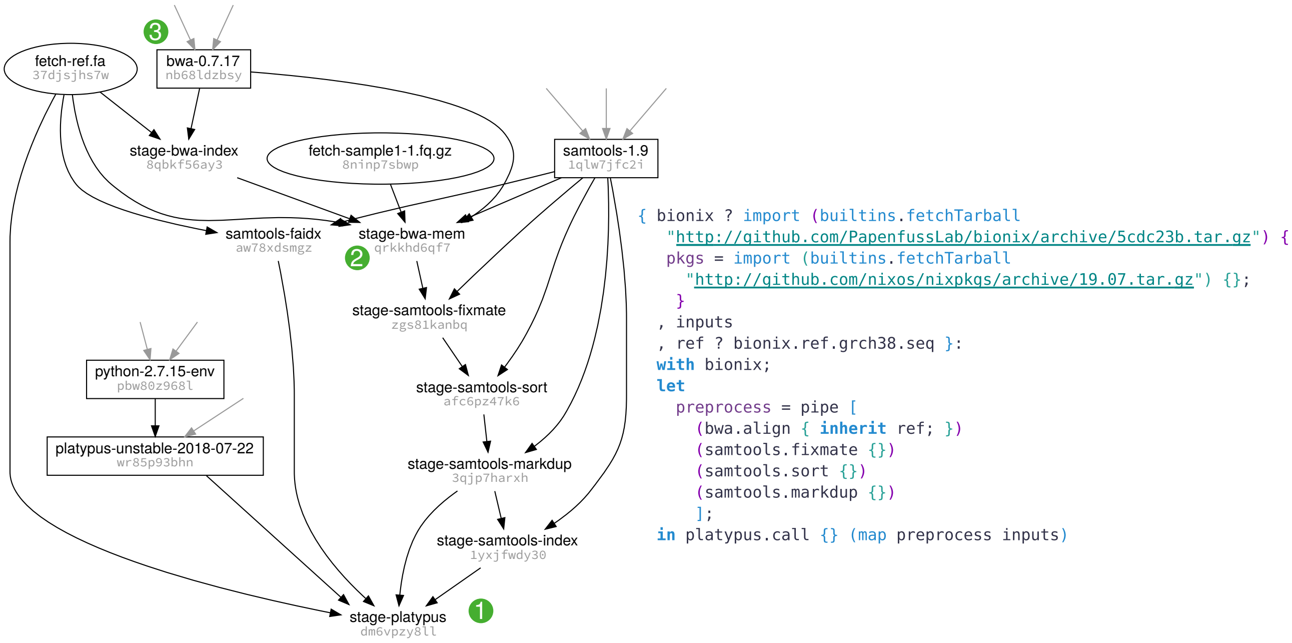


```
{ bionix ? import (builtins.fetchTarball
    "http://github.com/PapenfussLab/bionix/archive/5cdc23b.tar.gz") {
    pkgs = import (builtins.fetchTarball
      "http://github.com/nixos/nixpkgs/archive/19.07.tar.gz") {};
    }
  , inputs
  , ref ? bionix.ref.grch38.seq }:
  with bionix;
  let
    preprocess = pipe [
      (bwa.align { inherit ref; })
      (samtools.fixmate {})
      (samtools.sort {})
      (samtools.markdup {})
      ];
  in platypus.call {} (map preprocess inputs)
```

Example 1. An example workflow specified in BioNix (right) with a portion of the resulting build graph (left). In the build graph, rectangular nodes correspond to software dependencies and elliptical nodes to data files. Grey arrows indicate dependencies that are not illustrated in the figure. The workflow on the right corresponds to the terminal node in the build graph annotated with (1). The node annotated (2) in the graph corresponds to a single stage in workflow and the corresponding BioNix code can be found in Example 2. The final node annotated (3) corresponds to a software dependency provided by `nixpkgs` and the corresponding code is shown in Example 3.

computational environment. Dependencies are tracked down to the kernel level, and each stage is executed in its own sandbox, resulting in strong reproducibility guarantees and obviating the need for containers.

iii. Nix tracks the entire tree of runtime and build time dependencies with *fine grained versioning*. In the example pipeline this means that not only the version of `bwa`, but also the specific versions of `gcc` and `bash` under which `bwa` was compiled, are captured. All of these versions are fixed simply by specifying which versions of BioNix and `nixpkgs` we use (by their commit hashes): the code on the right forms a fully reproducible specification of the associated workflow. At the same time, it is straightforward to specify specific versions of software for distinct stages, and to use distinct versions of a given piece of software in parallel.

iv. BioNix uses *a simple, purely functional domain-specific language* – the Nix expression language – for specifying workflows. The Nix expression langauge has neither the inflexible verbosity of static configuration files, nor the error-prone power of a general-purpose programming language. Constructing a workflow is reduced to function composition; stages, workflows, and software dependencies are all represented as pure functions from dependencies to outputs. Because of purity, stages are guaranteed not to influence each other except through their inputs and outputs, and so can be safely recombined. An example of this in Example 1 is our ability to compose workflow steps using the higher-order `map` and `pipe` functions.

BioNix includes many features found among the most powerful existing workflow managers. Intermediate files do not need to be named or managed. Multiple versions of the same piece of software can be used simultanously. BioNix workflows are automatically parallelisable, can be executed in HPC environments or in the cloud, and are fully resumable in cases of interrupted execution.

BioNix includes the following components over and above base Nix:

i. A framework for specifying workflows in the Nix expression language.
ii. A library containing some commonly used bioinformatics tools and helpful workflow specification utilities.
iii. A module allowing workflows to be executed on HPC clusters.
iv. Basic typing to capture metadata and prevent invalid workflow specifications.

The rest of the paper first explains the basics of the Nix system and associated expression language. Next, we describe the design and implementation of BioNix. Finally, we describe an example workflow, and compare Bionix with existing bioinformatics workflow managers.

Preliminaries

The Nix deployment system

The Nix deployment system emerged from the work of Dolstra [9] and Dolstra, Jonge, and Visser [10]. Nix was originally designed as a software package manager, but has since been adapted to managing OS configurations (the `NixOS` project [11, 12]). BioNix represents a further extension of Nix to manage bioinformatics workflows.

The Nix system has three main components:

i. *Outputs* or *build products* may be any kind of directory, file, or collection of files. When using Nix as a traditional package manager, the build products typically consist of the compiled binaries and libraries associated with an application. In our case, build products are any output associated with a bioinformatics workflow or stage.

ii. *Derivations* are static configuration files (ending in `.drv`) that specify all of the build inputs and procedures required to produce a given build product. If a build product has prerequisites, then its derivation will refer to the derivations corresponding to those prerequisites.
iii. *Nix expressions* are written in a simple, high-level domain-specific language designed for specifying and manipulating "sets", which can be thought of as collections of name-value pairs (i.e., associative arrays).
Derivations are represented as "sets" (key-value pairs) in the Nix language, and are constructed by evaluating Nix expressions.
Unlike static configuration file formats such as YAML and JSON, the Nix expression language supports (pure) functions, which means that build processes and dependencies can be specified at a high level and in a way that minimises repetition. Nix expressions may also make use of various built-in design patterns to provide further extensibility and flexibility.

The basic build process in Nix is as follows: a Nix expression is *instantiated* to yield a tree of *derivations* describing how to generate the associated *build products*. Derivations are then *realised* by the build system to produce the build products themselves.

The Nix expression corresponding to a given build product will generally take the form of a pure function from dependencies to the corresponding output derivation. Using ML-style notation for types, one can represent this as

$$\text{Dependencies} \rightarrow \text{Output}.$$

Nix ensures that derivations are precisely specified by giving both derivations and build products hash-based names. The hash of a derivation is a function of all of the steps required to produce the associated build product, as well as the hashes of all of its dependencies.

The *Nix store*, usually located on the filesystem at `/nix`, provides a single, flat namespace for all derivations and build products and is writable by only the Nix system. Users typically access the store through *environments*: organised collections of soft links exported to `$PATH`.

The Nix community maintains online repository of prebuilt software called `nixpkgs` [13], which currently contains over 40,000 software packages.

The Nix expression language

We briefly introduce those parts of the Nix expression language required to understand the rest of the paper.

Sets are the most important datatype in Nix, and correspond to what are sometimes called associative arrays, records, or dictionaries in other languages. Set elements can be accessed by name:

Lists are delimited by square brackets and may contain elements of heterogeneous types separated by whitespace – for example,

The Nix language makes heavy use of *anonymous functions* (also called *lambda expressions*). The following denotes a Nix does not support functions of multiple arguments; instead, it is common for functions to take a set as input. Nix allows defaults to be provided for some elements, which are used if the function is called without providing the element. This is denoted using a question mark: the function `5`. Alternatively, one can mimic multi-argument functions using *higher-order* functions – i.e., functions which return functions. For example,

construction. We could bind the example above to a name Function application is denoted with whitespace (with lower precedence than list *Pattern matching* allows simultaneous binding of `x` into scope in the subsequent expression.

Implementation

The BioNix library itself is designed as a tree of functions, with each function representing one *stage* of processing. The BioNix tree is analogous to `nixpkgs`, with bioinformatics software (e.g., `bwa`, `samtools`, etc) forming the top level, and stages based on subcommands forming the second level (e.g., `bwa.align`). The BioNix tree is designed around the extensible design pattern of `nixpkgs`, allowing defaults to be overridden throughout the whole tree easily.

We will step through three examples of (slightly simplified) BioNix code that generates the build graph in Example 1: the workflow specification, a stage specification, and an expression for a software dependency.

Specifying a workflow

Example 1 shows a simple variant calling workflow using BWA [14, 15] for alignment, samtools [16] for sorting and duplicate marking, and platypus [17] for variant calling. The whole workflow is a single anonymous function, taking dependencies and inputs – the set spanning the first 8 lines – to an output (the final line):

$$\text{(Inputs, Options, \& Dependencies)} \rightarrow \text{Output}.$$

The output of this workflow is the output of platypus, which is a `.vcf` file.

One of the dependencies of the workflow is BioNix itself. If the user does not specify a version to use, the workflow defaults to using the specific commit indicated. Similarly, if the user does not specify a reference, the workflow defaults to GRCh38. Fixing a version of BioNix automatically fixes versions of all software used in the pipline.

Each stage – for example, `bwa.align`, `samtools.fixmate`, or `platypus.call` – is represented by a higher-order function that takes options and dependencies, and returns a function from inputs to outputs. The type of a stage[2] can be represented as

$$\text{(Options \& Dependencies)} \rightarrow \text{(Inputs} \rightarrow \text{Output)}.$$

BioNix dependencies by default include the BioNix tree itself to allow use of other (sub-)stages as well as the `nixpkgs` collection, which provides the required software prerequisites. For most of our stages we do not pass in any additional options, and so the first However, `bwa.align` requires that we specify a reference, and so we explicitly pass in the `ref` declared at the beginning of the workflow.

We make use of several helpful abstractions from functional programming. For example, we define a new function, `preprocess`, that takes a sample and performs alignment, mate-fixing, sorting, and duplicate-marking. We also use the `pipe` function in BioNix to sequentially compose a list of functions. Finally, we `map` this function over all our inputs. The Nix expression language allows for this abstraction and modularity without introducing side-effects.

2 Functional programmers will recognise this as a *curried* version of the type of a workflow.

Specifying a stage

```
{ bionix
, ref
, bamOutput ? false}:

{ input1
, input2 ? null}:

with nixpkgs;
with lib;

stdenv.mkDerivation {
  name = "bwa-mem";
  buildInputs = with pkgs; [ bwa samtools ];
  buildCommand = ''
    ln -s ${ref} ref.fa
    for f in ${bionix.bwa.index {} ref}/* ; do
      ln -s $f
    done

    bwa mem -t $NIX_BUILD_CORES \
      ref.fa \
      ${optionalString (input2 != null) (input2)} \
      ${optionalString bamOutput "| samtools view -b"}
  '';
}
```

Example 2. Specifying an alignment stage using BWA-mem.

Example 2 illustrates an example *stage* in BioNix. In line with our design pattern, the whole stage is represented by an anonymous higher-order function: it takes a record of options and dependencies, and returns a function that takes inputs – in this case, a pair of FASTQ files representing read pairs – and returns a derivation. Notice that we give the reference as part of the first argument to the stage (options and dependencies) rather than as part of the second argument (inputs). This is because often an entire workflow will be parametrised by a single reference genome.

Links are created for both the reference and its associated BWA indices to deal with the standard bioinformatics convention that indices are located in the same directory as the associated indexed file.

Finally, the output is optionally converted to the `.bam` format within the shell script associated with the derivation. BioNix cannot stream data between stages of a workflow: both inputs and outputs of a stage must be a file or set of files.

Specifying a software dependency

For completeness, we consider how to specify a software dependency. In our example workflow, the BWA software is provided by `nixpkgs` and Example 3 shows a simplified version of its specification there.

i. The expression is an anonymous function from dependencies – in this case, the utility libraries `stdenv` and `fetchurl` and the C library dependency `zlib` – to outputs – in this case, the compiled binary for `bwa`.
ii. The function body is just a single call to the helper function `mkDerivation`. Since `bwa` follows the first two parts of the common `./configure; make; make install` pattern for building unix software, only the final install phase needs to be specified. Here, the resulting binary is copied into the `bin/` directory.

```
{ stdenv, fetchurl, zlib } :

stdenv.mkDerivation rec {
  name = "bwa-${version}";
  version = "0.7.17";

  src = fetchurl {
    url = "mirror://sourceforge/bio-bwa/${name}.tar.bz2";
    sha256 = "1zfhv2zg9v1icdlq4p9ssc8k01mca5d1bd87w71py2swfi74s6yy";
  };

  buildInputs = [ zlib ];

  installPhase = ''
    install -vD bwa $out/bin/bwa
  '';
}
```

Example 3. Specifying a software dependency for bwa-mem. This is a simplified version of the expression found in `nixpkgs`.

HPC queue integration

While the Nix build system provides support for both local and remote building, bioinformatics workflows are commonly executed on traditional High Performance Computing (HPC) infrastructure managed by a job schedulers. These systems require users to submit jobs to a queue, along with specified resource limits.

BioNix provides support for queuing systems via a function that takes resource limits and a derivation, and returns a new derivation that will submit the build process as a job to the queuing system instead of building it directly. This design allows arbitrary derivations to be lifted to the queue, and also allows users to combine submission to the queue and building via the Nix build system directly. However, as submission is a (relatively benign) side effect, builds cannot be realised using sandboxing.

Tracking types of build products

BioNix gives build products optional *types* in order to prevent some errors in workflow specification and to track useful metadata such as the reference used for an alignment. This is a lightweight version of the approach taken by *Bioshake* [18, 19]. Types are implemented as an Abstract Data Type (ADT) and are tracked using Nix's passthru features.

Discussion

Real world use of BioNix

Small variant calling workflow. We have used BioNix to manage a workflow that performs somatic variant calling and Copy Number Variant (CNV) calling on whole genome deep sequencing human data using BWA [14, 15] for alignment, samtools [16] for sorting and marking duplicates, Strelka [20] for somatic variant calling, and CNVkit [21] for CNV calling.

This workflow was executed on HPC infrastructure managed with the TORQUE resource manager [22] using the extensions presented earlier. A total of 1.1TB of (compressed) fastq input was processed, producing 755GB of results (including alignments).

We also implemented an extension allowing the automatic generation of reports, standardising our analysis and reporting of routine samples. The report is generated from a text template with tables and sections populated by Nix build products. In this way, a complete analysis is captured and rendered highly reproducible: a report can always be recreated from the input FASTQ files using a fixed set of software.

Structural variant calling at scale. We have also used BioNix to execute a workflow that processes 6.8TB of whole genome sequencing data from mice, performing quality checking, alignment, and merging, and structural variant calling using `gridss` [23] with a range of parameters. This resulted in a total of 5.3TB of results.

Related work

We discuss here two categories of work related to our own. The first consists of other projects making use of the Nix deployment system to manage data processing workflows; the second concerns existing workflow management tools popular in bioinformatics and computational biology.

Similar adaptations of the Nix system

Several groups have made use of Nix to manage the *environments* in which computational workflows are executed. Researchers at GRICAD at the Université Grenoble Alpes have made use of Nix as an HPC package management system [24, 25]. The Pipelines in Genomics (PiGx) project [26] uses Guix – an implementation of the Nix system using GNU Scheme in place of the Nix expression language – to produce a set of reproducible "turn-key" workflows for bioinformatics and computational biology, configured via simple static config files. Similar uses of Nix for reproducible research have also been suggested by Blair Archibald of the Software Sustainability Institute [27, 28] and Bruno Vieira at the Mozilla Foundation [29].

However, none of these approaches use Nix to specify workflows themselves; instead, Nix is used as a replacement for package managers and containers. BioNix takes the next step and embeds the workflows into the Nix system.

Two projects that we know of make use of Nix to manage workflows themselves: Mix, a Nix-based system for specifying data processing pipelines developed at SoundCloud [30], and Fractalide, a service programming platform using dataflow graphs [31].

Mix is built on the `hnix` project [32] and implements a new builder dedicated to data workflows. Mix redefines derivations to remove the Nix store and allow storage of products on a distributed file system. Consequently, Mix cannot take advantage of `nixpkgs` and focuses entirely on the workflow without capturing the computational environment. In contrast, BioNix captures the workflow along with the software environment required to execute it.

Fractalide is an effort to provide a dataflow graph programming platform with an initial focus on microservices and the internet of things. Though it builds on Nix, it also extends the base language with a new language for specifying the dataflow graphs, and relies on various language bindings to provide an interface to the actual data processing (i.e., the microservice). By contrast, BioNix focuses on Bioinformatics workflows and is implemented entirely within the existing Nix ecosystem as a lightweight extension, and relies on calling existing software via their existing command line interfaces.

Existing workflow managers for computational biology

As already mentioned, current best practice aims at reproducibility using a combination of package managers, containers, and workflow engines. BioNix replaces all of these, and so is in some sense difficult to compare with existing workflow management tools.

However, we can compare the syntax of BioNix with that of existing workflow managers by implementing toy piplines in each. Examples 4 and 5 illustrate two simple examples from the documentation of Workflow Description Language (WDL) [33] and NextFlow [34] alongside the equivalent BioNix expression. Unlike the WDL and NextFlow examples, BioNix captures the software used in the execution of the workflow. As workflows in BioNix are simple function compositions, the final workflow definition is more concise than the equivalent WDL fragment. Nextflow workflow specification is more implicit than in BioNix or WDL, which may increase the difficulty of interpreting and maintaining complex workflows.

BioNix might also be compared with Common Workflow Language (CWL) [37], which is a low-level specification language intended for describing workflows in a portable way. However, CWL specifications increasingly play a role similar to Nix's derivation files: derivations are a complete, portable description of builds that can be transferred, shared and built on local or remote systems by a build engine. However, derivations are not written directly as they are too low level, and BioNix provides functions to specify workflows at a high level. Similarly, CWL is quite low level and requires tooling to allow higher level specifications.

Galaxy [38] is a popular workflow platform that provides a web-based GUI for specification of workflows and execution controls. Galaxy does provide facilities to manage the computation environment via various package management tools, with Conda being popular. It is possible to integrate Nix into Galaxy, which would allow Galaxy to control the computational environment to a significantly greater degree than what is currently offered. This would be similar to PiGx and has the same disadvantages already discussed.

Cuneiform [39, 40] is a functional programming language for large-scale data analysis workflows. In Cuneiform, as in BioNix, workflow stages are modelled as pure functions. Cuneiform also has an elegant foreign function interface (FFI), allowing the seamless use of code snippets from a variety of languages – bash, Python, R, and others – as well as a language-level static type system. On the other hand, Cuneiform doesn't manage software dependencies, and so lacks the reproducibility guarantees that BioNix leverages from Nix.

The Guix Workflow Language (GWL) [41, 42] is in many ways the workflow manager closest in approach to BioNix. GWL, like PiGx, is built on Guix, and so inherits the reproducibility guarantees of a Nix-like system. Moreover, unlike PiGx, GWL manages workflows themselves using Guix, rather than using it only to provide the necessary software environment. However unlike BioNix, stages in GWL are not represented by functions but by data structures, workflows are specified via manual construction of the associated build graph, and no types are used to prevent construction errors.

Conclusions

We have presented BioNix, a framework built on Nix in which workflows are specified using pure functions. BioNix captures software versions and dependencies, manages computational environments, and composes the various stages of workflows all within the one framework and language. Previous approaches to computational reproducibility have relied on a combination of technologies such as containers, package managers, and workflow engines to achieve the same ends. BioNix unites these functionalities under the one framework, making computational biology workflows easier to specify, share, execute, modifying, and reproduce.

BioNix is available at http://github.com/PapenfussLab/bionix under the 3-clause BSD license.

```
{ bionix ? import <bionix> {} }:

with bionix;
with lib;

let

  prepare = splitString "\n" (removeSuffix "\n" (readFile (stage {
    name = "prepare";
    buildInputs = [ pkgs.python3 ];
    buildCommand = ''
      python -c "print('one\ntwo\nthree\nfour', end='''')" > $out
    '';
  })));

  analysis = str: removeSuffix "\n" (readFile (stage {
    name = "analysis";
    buildInputs = [ pkgs.python ];
    buildCommand = ''
      python -c "print('_${str}_')" > $out
    '';
  }));

  gather = strs: stage {
    name = "gather";
    buildCommand = ''
      echo ${concatStringsSep " " strs} > $out
    '';
  };

in gather (map analysis prepare)
```

```
task prepare {
  command <<<
    python -c "print('one\ntwo\nthree\nfour')"
  >>>
  output {
    Array[String] array = read_lines(stdout())
  }
}

task analysis {
  String str
  command <<<
    python -c "print('_${str}_')"
  >>>
  output {
    String out = read_string(stdout())
  }
}

task gather {
  Array[String] array
  command <<<
    echo ${sep=' ' array}
  >>>
  output {
    String str = read_string(stdout())
  }
}

workflow example {
  call prepare
  scatter (x in prepare.array) {
    call analysis {input: str=x}
  }
  call gather {input: array=analysis.out}
}
```

Example 4. Scatter-gather example from WDL [35] documentation with the BioNix implementation on the left and WDL on the right. Unlike WDL, the python software is explicitly specified as a build requirement. The BioNix example also demonstrates how different versions of software can be mixed: the prepare stage uses Python 3 while the analysis stage relies on Python 2.

```
{ bionix ? import <bionix> {}
, input ? ./sample.fa}:

with bionix;
with lib;

let
  splitSequences = fa: stage {
    name = "splitSequences";
    buildInputs = [ pkgs.gawk ];
    buildCommand = ''
      awk '/^>/{f="seq_"++d} {print > f}' ${fa}
      mkdir $out
      cp seq* $out
    '';
  };

  reverse = fa: stage {
    name = "reverse";
    buildCommand = ''
      ${pkgs.utillinux}/bin/rev ${fa} > $out
    '';
  };

in pipe [
  splitSequences
  (each reverse)
] input
```

```
params.in = "$baseDir/data/sample.fa"
sequences = file(params.in)

/*
 * split a fasta file in multiple files
 */
process splitSequences {

    input:
    file 'input.fa' from sequences

    output:
    file 'seq_*' into records

    """
    awk '/^>/{f="seq_"++d} {print > f}' < input.fa
    """

}

/*
 * Simple reverse the sequences
 */
process reverse {

    input:
    file x from records

    output:
    stdout result

    """
    cat $x | rev
    """
}

/*
 * print the channel content
 */
result.subscribe { println it }
```

Example 5. Nextflow basic pipeline example [36] (right) translated to BioNix (left). The BioNix expression requires specification of the software used, and hence GNU Awk is chosen here. The Nextflow pipeline does not capture the software used. The BioNix `pipe` function implements reverse function composition allowing a natural specification of workflows.

Acknowledgements

Thanks to Ramyar Molania and Jocelyn Sietsma Penington for being early adopters of BioNix, and to Ramyar for helpful comments on the manuscript. Thanks to Alan Rubin for many helpful discussions and comments on the manuscript.

A.T.P. was supported by the Lorenzo and Pamela Galli Charitable Trust and by an Australian National Health and Medical Research Council (NHMRC) Program Grant (1054618) and NHMRC Senior Research Fellowship (1116955). The research benefitted by support from the Victorian State Government Operational Infrastructure Support and Australian Government NHMRC Independent Research Institute Infrastructure Support.

J.B. was supported by the Stafford Fox Medical Research Foundation.

Competing interests

The authors declare that they have no competing interests.

References

1. Reality check on reproducibility. Nature 2016;533:437–7.
2. Challenges in irreproducible research. Nature, 2018.
3. Package, dependency and environment management for any language—Python, R, Ruby, Lua, Scala, Java, JavaScript, C/ C++, FORTRAN. 2018. URL: https://conda.io/docs/.
4. Enterprise container platform. 2018. URL: https://www.docker.com.
5. Singularity. 2018. URL: https://www.sylabs.io/singularity/.
6. Vivian J, Rao AA, Nothaft FA, et al. Toil enables reproducible, open source, big biomedical data analyses. Nature Biotechnology 2017;35:314–6.
7. Goodstadt L. Ruffus: a lightweight Python library for computational pipelines. Bioinformatics 2010;26:2778–9.
8. Grüning B, Chilton J, Köster J, et al. Practical Computational Reproducibility in the Life Sciences. Cell Systems 2018;6:631–5.
9. Dolstra E. The Purely Functional Software Deployment Model. PhD thesis. Faculty of Science, Utrecht, The Netherlands, 2006.
10. Dolstra E, Jonge M de, and Visser E. Nix: A Safe and Policy-Free System for Software Deployment. In: *Proceedings of the 18th Large Installation System Administration Conference* (Atlanta). 2004.
11. Dolstra E, Löh A, and Pierron N. NixOS: A Purely Functional Linux Distribution. Journal of Functional Programming 2010:577–615.
12. NixOS. 2019. URL: https://www.nixos.org/nixos.
13. nixpkgs. 2019. URL: https://www.nixos.org/nixpkgs.
14. Li H. Aligning sequence reads, clone sequences and assembly contigs with BWA-MEM. 2013. arXiv: q-bio/1303.3997.
15. Li H and Durbin R. Fast and accurate short read alignment with Burrows-Wheeler transform. Bioinformatics 2009;25:1754–60.
16. Li H, Handsaker B, Wysoker A, et al. The Sequence Alignment/Map format and SAMtools. Bioinformatics 2009;25:2078–9.
17. Rimmer A, Phan H, Mathieson I, et al. Integrating mapping-, assembly- and haplotype-based approaches for calling variants in clinical sequencing applications. Nature Genetics 2014;46:912–8.
18. Bedő J. Bioshake: a Haskell EDSL for bioinformatics pipelines. 2018. bioRxiv: http://doi.org/10.1101/529479.
19. Bioshake. 2019. URL: https://github.com/PapenfussLab/bioshake.
20. Kim S, Scheffler K, Halpern AL, et al. Strelka2: fast and accurate calling of germline and somatic variants. Nature Methods 2018;15:591–4.
21. Talevich E, Shain AH, Botton T, and Bastian BC. CNVkit: Genome-Wide Copy Number Detection and Visualization from Targeted DNA Sequencing. PLOS Computational Biology 2016;12:e1004873.
22. TORQUE Resource Manager. 2019. URL: http://www.adaptivecomputing.com/products/torque/.
23. Cameron DL, Schröder J, Penington JS, et al. GRIDSS: sensitive and specific genomic rearrangement detection using positional de Bruijn graph assembly. Genome Research 2017;27:2050–60.
24. Bzeznik B, Henriot O, Reis V, Richard O, and Tavard L. Nix as HPC package management system. In: *Proceedings of the Fourth International Workshop on HPC User Support Tools - HUST'17*. the Fourth International Workshop. Denver, CO, USA: ACM Press, 2017:1–6. DOI: 10.1145/3152493.3152556.
25. Bouttier PA. Nix as HPC package management system. NixCon. 2018.
26. Wurmus R, Uyar B, Osberg B, et al. PiGx: reproducible genomics analysis pipelines with GNU Guix. GigaScience 2018;7.
27. Archibald B. Reproducible Environments With Nix. Software Sustainability Institute. 2017. URL: https://www.software.ac.uk/blog/2017-10-05-reproducible-environments-nix (visited on 01/23/2019).
28. Crouch S, Hong NC, Hettrick S, et al. The Software Sustainability Institute: Changing Research Software Attitudes and Practices. Computing in Science Engineering 2013;15:74–80.
29. Vieira B. A truly reproducible scientific paper? Bruno Vieira. 2017. URL: https://medium.com/@bmpvieira/a-truly-reproducible-scientific-paper-5059b282ee9a (visited on 01/23/2019).
30. Georges Dubus. Mix: Nix for data pipeline configuration. NixCon. London, 2018.
31. Reusable Reproducible Composable Software. 2019. URL: https://github.com/fractalide/fractalide.
32. A Haskell re-implementation of the Nix expression language. 2019. URL: https://github.com/haskell-nix/hnix.
33. WDL | Home. URL: https://software.broadinstitute.org/wdl/ (visited on 01/23/2019).
34. Di Tommaso P, Chatzou M, Floden EW, Barja PP, Palumbo E, and Notredame C. Nextflow enables reproducible computational workflows. Nature Biotechnology 2017;35:316–9.
35. Workflow Description Language - Specification and Implementations. 2019. URL: https://github.com/openwdl/wdl#scattergather.

36. Nextflow - Basic pipeline. 2019. URL: https://www.nextflow.io/example1.html.
37. Amstutz P, Crusoe MR, Tijanić N, et al. Common Workflow Language, v1.0. 2016. DOI: 10.6084/m9.figshare.3115156.v2.
38. Afgan E, Baker D, Batut B, et al. The Galaxy platform for accessible, reproducible and collaborative biomedical analyses: 2018 update. Nucleic Acids Research 2018;46:W537–W544.
39. Brandt J, Bux M, and Leser U. Cuneiform: A Functional Language for Large Scale Scientific Data Analysis. In: *Proceedings of the Workshops of the EDBT/ICDT*. Vol. 1330. Brussels, Belgium, 2015:17–26.
40. Brandt J, Reisig W, and Leser U. Computation semantics of the functional scientific workflow language Cuneiform*. Journal of Functional Programming 2017;27.
41. Janssen, Roel. Workflow management with GNU Guix. FOSDEM 2017. 2017.
42. Wurmus R. GWL: GNU Workflow Language. FOSDEM 2019. 2019.
