# Supplementary material for: Unifying package managers, workflow engines, and containers: Computational reproducibility with BioNix
Source: Gigascience. 2020 Nov 18;9(11):giaa121. doi: 10.1093/gigascience/giaa121 (PMC7672450; doi:10.1093/gigascience/giaa121)
Supplement: giaa121_GIGA-D-19-00324_Revision_1 [file giaa121_giga-d-19-00324_revision_1.pdf]

GigaScience

Unifying package managers, workflow engines, and containers: computational reproducibility with BioNix

--Manuscript Draft--

**Manuscript Number:**	GIGA-D-19-00324R1
**Full Title:**	Unifying package managers, workflow engines, and containers: computational reproducibility with BioNix
**Article Type:**	Technical Note
**Funding Information:**	Australian National Health and Medical Research Council (1054618) — Dr Anthony T. Papenfuss<br>Australian National Health and Medical Research Council (1116955) — Dr Anthony T. Papenfuss
**Abstract:**	Motivation: A challenge for computational biologists is to make our analyses reproducible -- that is, easy to rerun, combine, and share, with the assurance that equivalent runs will generate identical results. Current best practice aims at this using a combination of package managers, workflow engines, and containers.<br><br>Results: We present BioNix, a lightweight library built on the Nix deployment system. BioNix manages software dependencies, computational environments, and workflow stages together using a single abstraction: pure functions. This lets us specify workflows in a way that is more reproducible and modular than current best practices.<br><br>Availability and implementation: BioNix is implemented in the Nix expression language and is released on GitHub under the 3-clause BSD license: https://github.com/PapenfussLab/bionix.
**Corresponding Author:**	Justin Bedo<br><br>AUSTRALIA
**Corresponding Author Secondary Information:**	
**Corresponding Author's Institution:**	
**Corresponding Author's Secondary Institution:**	
**First Author:**	Justin Bedo
**First Author Secondary Information:**	
**Order of Authors:**	Justin Bedo

Powered by Editorial Manager® and ProduXion Manager® from Aries Systems Corporation

	Leon Di Stefano
	Anthony T. Papenfuss
**Order of Authors Secondary Information:**	

Response to Reviewers:

We thank the editor and reviewers for very useful feedback. We have prepared a revised version that addresses the issues raised and strengthens the manuscript. We respond to the individual points in-line.

Editor

> I wish to highlight the comment of reviewer 2 who feels that more
> extensive life-science use cases should be included (in a reproducible
> way, including the data). Open, real-life use cases that demonstrate
> the tool in action are indeed an important feature of our "Technical
> Note" section.

We have added examples to the paper of actual workflow descriptions used, and provided a full real-world example in the repository that processes whole-genome data in a somatic tumour-normal analysis.

> Reviewer 3 feels the presentation of advantages and disadvantages of
> your and similar tools was biased in the present version of the paper.
> I do note that reviewer 3, Johannes Köster, is a co-author of
> "Snakemake", a competitor tool. However, generally speaking, I agree
> with the reviewer's advice that statements on a tool's features and
> advantages/disadvantages should be as factual and unbiased as
> possible.

We apologise for the unintentional bias present and have rewritten the manuscript to add greater discussions around the limitations of our approach.

> Please also register your new software application in the bio.tools
> and SciCrunch.org databases to receive RRID (Research Resource
> Identification Initiative ID) and biotoolsID identifiers, and include
> these in the software availability section your manuscript. This will
> facilitate tracking, reproducibility and re-use of your tool.

We have registered BioNix in the bio.tools registry (biotools:BioNix) and SciCrunch database (SCR_017662). These are now cited in the manuscript.

> Please also ensure that your revised manuscript conforms to the
> journal style, which can be found in the Instructions for Authors on
> the journal homepage.

We have endeavoured to follow the journal style, and would be delighted to address any remaining style issues.

Reviewer #1:

> Novel approach to composition of bioinformatics workflows, targeted to
> developers. Some minor additions to the paper are required: 1). HPC
> environment is mentioned, and the integration with queuing systems,
> how are jobs in the workflow that are dropped by the queuing system
> are handled in bionix ?

We have added a discussion on queue failures to the HPC section, clarifying that queue errors are handled similarly to execution errors: the build is aborted.

> 2). A little more comparison with existing workflow systems is needed
> in the discussion section (there is only a brief mention of Galaxy,
> for the bionix integration with it). How does for example bionix
> compare with nextflow, snakemake etc ? A comparison with points on

> features (present / absent in bionix) to the other workflow platforms
> would be useful in the discussion, for readers looking to compare and
> decide which platform to adopt.

We have expanded the "comparisons" section and included a discussion of Nextflow, along with an explicit syntax comparison. We have also added a "limitations" section that explains the trade-offs involved in BioNix's approach.

We have shied away from a "feature matrix" comparing existing tools for three reasons.

First, our primary contribution—a system that unifies package managers, containers, and workflow tools, using the one abstraction—is less a new feature than novel approach to workflow specification, and we wanted to keep our paper focused on this. (Guix is the closest precedent.)

The second reason is that our approach makes some feature comparisons quite difficult. For example, it is possible to integrate Docker containers into BioNix workflows, and to export workflows in containerised form. But doing so sacrifices the modularity and isolation gained by working within the Nix ecosystem. As such, "Docker support" is not, for us, unambiguously a "feature".

Finally, BioNix itself is less a workflow management system than a thin layer adapting the Nix system to the needs of bioinformaticians. It inherits most of its features from Nix itself—a mature, independent project that is not bioinformatics specific.

> 3). Does bionix integrate with Docker and if not, any disadvantages
> from that ? I believe it should be considered as standard that modern
> bioinformatics workflow systems can run tools prepackaged in
> containers at any of the analysis steps included in the workflow (the
> use of containers is becoming pervasive in bioinformatics for multiple
> reasons). Look for example the nextflow platform, and also Galaxy
> which now enables tool execution from containers.

It is possible to integrate containers into a workflow, as nixpkgs has support for working with Docker and Singularity containers, conda packages, and even binaries. However, using these creates side effects: the construction of the container image is not captured in the Nix specification. Such side effects should be kept to a bare minimum in this system, so container use is discouraged except in the rare cases when no alternative is possible. Our motivating goal is after all to provide a framework that includes computational environment management, obviating the need for containers. We have added a clarifying footnote.

Reviewer #2

> The author describes BioNix, submitted as a technical note. The paper
> is well written, motivates the development well, and discusses
> relevant previous work. Also, we were able to build and run some
> simple examples locally on a Linux laptop. Despite being a technical
> note, my main concern is that we believe that the case studies
> presented in Life Science are very thin.
>
> == Major concerns
>
> Main The authors should present much more in-depth analysis of
> concrete applications in life science. Now the paper is almost
> exclusively a technical description, and the section "Real world use
> of BioNix" is very short and lacks details. No code is presented. If
> more and longer analyses on life science data are available then it
> could be accepted as a Technical Note in Gigascience. Else, I would
> consider this manuscript to be more suited to a more specialized
> technical journal. Further, with such a high focus on reproducibility,
> make sure the readers can reproduce all the cases in the "Real world

Powered by Editorial Manager® and ProduXion Manager® from Aries Systems Corporation

> use" section.

We have expanded the real-world section to include the workflow code used to process the examples given. We have not presented any results from the actual analysis of the data as we feel this is outside the scope of the paper.

For the tumour-normal small variant calling workflow, though we cannot release the data we have processed internally using the workflow (it comprises numerous clinical samples and is part of active treatment planning), we have put an example using publicly available melanoma data available from the ENA into the BioNix repository. These data represent a realistic use-case presented in the paper.

The second example workflow, consisting of structural variant calling in mice, has also been presented in the manuscript. However, as the project is unique we haven't been able to find suitable surrogate data to provide a fully executable example. We would be happy to add an additional structural variant calling step to the tumour-normal calling workflow showing how the structural variant calling step scales easily to real-world whole genomes if the reviewers deem it necessary.

> Apart from the main concern, also the following points need to be
> addressed:
>
> 1. There is no section discussing known limitations. All design
> desicions come with certain tradeoffs. Thus for clarity and fairness
> to the reader, we think such a section should be included.

We have added a section to the discussion on known limitations.

> 2. There is one area that could use some more elaboration: The
> handling of multiple inputs and outputs. This is a common case in many
> types of workflows, and in some cases two outputs from one process
> need to take different downstream routes, such as when splitting a
> dataset into a test- and a training set for machine learning.

We have added an example of picard tools producing multiple outputs (the BAM and metrics) to the section. Your example of test and training splits would fit into this with the training data forming the main output and the test data forming an additional output. The test data could then be referred to explicitly in a later stage.

> 3. The examples lack an overview describing what happens in each
> example. This makes it a bit hard to know what to expect from the
> examples.

We have expanded the captions to include an overview of the example in addition to more expository text.

> 4. An area that can often get cumbersome in some cases is where only
> the dependencies between stages or processes are defined, but not how
> each individual output are connected to downstream processes. A
> discussion of how this issue is handled in BioNix would be very
> helpful.
>
> 5. Consider citing the following highly relevant papers:
> - https://www.nature.com/articles/s41592-018-0046-7
> - https://academic.oup.com/gigascience/article/8/5/giz044/5480570

Thank you for the suggestions. We have added bioconda to the discussion of conda. We have also added a discussion comparing our work to scipipe to the manuscript.

> == Minor concerns
>
> Page 1, column 2, 5th row from bottom:

Powered by Editorial Manager® and ProduXion Manager® from Aries Systems Corporation

> "the one" perhaps should be "one"?
>
> Page 2, column 1, you say:
> "inflexible verbosity of static conguration files"
> - Is static configuration files really known for verbosity?
> Inflexibility, yes, but verbosity?

We have rewritten this comment to acknowledge the tradeoffs involved in using static configuration files vs. domain-specific languages vs. a general purpose programming language.

> Page 3, column 1, you say:
> "Alternatively, one can mimic multi-argument functions using higher-order functions - i.e., functions which return functions."
> - This is not very clear how that works, and an example or more elaborate explanation would help.

Apologies, that section of the manuscript inadvertantly changed and the example was missing. We have restored the example which should clarify things.

> Page 3, column 2: Broken sentence?
> "For most of our stages we do not pass in any additional options,
> and so the first However"

We have corrected this sentence.

> Page 4, column 2: This sentence needs a little more explanation:
> " However, as submission is a (relatively benign) side effect, builds cannot be realised using sandboxing."

We have clarified the statement.

> Page 5, column 2:
> "written directly"
> Should probably be:
> "written by humans"

We have updated the sentence.

> Page 5, column 2, under "Conclusions"
> "modifying" seems it should be "modify"

We have updated the sentence.

Reviewer #3

> The manuscript describes BioNix, a unified workflow management system
> that uses the nix framework to integrate package management with
> workflow management. The central innovation of BioNix that it finds a
> way to express both the workflow steps as well as software deployment
> steps via the same, functional, Nix DSL.
>
> The paper is written in an understandable and well structured way. The
> central innovation mentioned above is an interesting addition to what
> is available in the field, and indeed deserves to be published.
> Unfortunately, the paper in its current form, in particular when
> comparing with other approaches is sometimes unnecessarily biased and
> not properly justified. It seems like the manuscript tries to convince
> the reader that the presented approach is superior to others. However,
> this claim is never more than a plain hypothesis, as any given
> arguments are not convincing. Instead, I suggest to simply present the
> new approach and maybe briefly compare to others without making a
> subjective judgement. Please find details below.

We have substantially changed the tone of our paper in line with this suggestion. See below for details.

Powered by Editorial Manager® and ProduXion Manager® from Aries Systems Corporation

> # Major comments
>
> The introduction correctly lists three pillars of in silico
> reproducibility. However, there is more to it. First, scalability is
> an issue as well. People need to be able to scale a workflow to their
> computational environment (e.g. cluster or cloud) ideally without the
> need to modify the workflow definition itself. Second, transparency
> and readability is important as well. Apart from computational
> reproduction as a black box, it is important that people can judge
> over the taken steps in terms of parameters and code. Hence, it is
> important that a workflow is in large parts human readable without
> knowing the used definition language.

Thank you for pointing out an interesting aspect. We agree that scalability is paramount, and that the definition of a workflow needs to be both readable and independent of how the execution takes place. We have added a discussion of this to the introduction.

That said, we feel that scalability is a consequence of reproducibility (and compositionality) rather than a component of it: when the stages of a workflow are easily reproduced anywhere—e.g., remotely, in the cloud, or as part of a computing queue—the workflow is inherently scalable.

On readability: we believe that modelling workflows with simple function composition substantially increases readability. That said, claims about readability can be quite subjective, and dependent on the programming languages and paradigms that users are already familiar with. We believe that our side-by-side syntax comparisons, and our "limitations" section, now adequately address this.

> Page 2: sandboxing is indeed a nice feature, which, as the authors
> correctly conclude, can also be achieved with containers. The authors
> should clarify whether it has to be enabled/disabled globally or if it
> is possible to change this per step. Also, they should highlight more
> prominently that BioNix only supports it for local (or cloud?)
> execution, not for cluster execution.

We have clarified that the sandboxing can be disabled either globally or per build. We have also clarified the no-sandbox restriction only applies to cluster execution and that remote building fully supports sandboxes.

> Page 2: it is first mentioned that the nixpkgs import fixes the
> versions of all used software, and later the authors mention that
> software versions can be defined per stage. The authors should provide
> an example of the latter.

We have added examples 2 and 3 to the manuscript demonstrating a couple of ways that software can be explicitly versioned.

> Page 2, paragraph 3: the statement "inflexible verbosity of
> configuration files ... error-prone power of general purpose language"
> is fairly judgemental, without providing any reasoning. If the authors
> make such a statement, they need to provide particular reasons in
> detail, and also highlight the upsides of both. Also, an aspect not
> considered here is that there are situations where config files are
> more suitable than a functional language and as well where a general
> purpose programming language is more suitable than a functional
> specification language like Nix. One could argue that this is the case
> for workflow management. It combines the need for declaring options,
> parameters, software dependencies (which configuration file languages
> are designed for), with the need for declaring steps (obviously a
> declarative task) and the need for flexibility in certain corner
> cases, e.g. complex aggregations, conditional execution (for which the
> full power of general purpose programming languages might be needed).
> Hence, there is certainly a case for not unifying all the different

Powered by Editorial Manager® and ProduXion Manager® from Aries Systems Corporation

> aspects of workflow definition in a single language. I would like to
> see the paper discussing this openly, without making a judgement. An
> example for such a system is Snakemake, which provides a declarative
> syntax for workflow step definition on top of Python, a general
> purpose language, in combination with YAML for configuration files and
> conda software environment definition files for ensuring controlled
> execution environments. Since Snakemake is among the most widely used
> workflow managers it should definitely be discussed along with Galaxy,
> Nextflow, WDL and CWL here.

We have expanded our discussion of the tradeoffs involved in using configuration files vs. a domain specific language vs. a general purpose programming language.

> If understood correctly, the submission of a BioNix workflow to a
> cluster system needs the modification of the workflow definition by
> adding submission functions. This would limit reproducibility (and
> should be communicated as such), because the workflow potentially
> needs to be adapted before execution on a new system.

We have made it clear that this modification can take place through a single, global override. The tumour-normal small variant calling workflow in the repository now contains an example of this for submission to torque or slurm.

We believe that including job submissions in the workflow in this way lets us both 1) be "up front" about potential side effects associated with choosing a given job submission system; while at the same time 2) allowing queueing systems to be switched easily and globally.

> The comparison with other systems is extremely biased. See details
> below.

> For example, both Snakemake and Nextflow support streaming between
> jobs and conditional execution, which does not seem to be supported by
> BioNix. It is of course infeasible to list the union of all features
> of all workflow management systems, but (a) this should be clearly
> communicated and (b) one should try to find a balance of advantages
> and disadvantages of all systems, or even better just list available
> and missing features and leave the judgement to the reader.

We have now stated explicitly that BioNix does not support streaming between stages.

BioNix does support conditional execution, and we have made this clear in the main text.

> The authors mention that the nextflow definition is more implicit than
> BioNix, and this may increase difficulty of interpretation. First,
> from the example, I cannot see this increased implicitness. Second,
> implicitness can also be an advantage, and explicitness also has
> downsides. Please communicate this openly.

We have removed this comment.

> I could not follow the comparison with CWL. First, I do not agree that
> CWL is low-level. I would agree with a statement saying that CWL is
> quite verbose and hard to read. However, at least for somebody not
> familiar with Nix, this is also the case for BioNix. Readability for
> non-authors is a very important aspect, and both systems are not the
> best I have seen so far in this regard.

We have removed the labelling of CWL as low-level, but have clarified in the text that CWL is increasingly used as a machine-readable target by other build systems, and this sense is more analgous to Nix's derivation format than to BioNix per se.

Powered by Editorial Manager® and ProduXion Manager® from Aries Systems Corporation

> The statement that Nix integration in Galaxy would lead to a better
> control of the computational environment has to be justified. What
> makes Nix better than Conda or Containers, in terms of control?

Because of its hash-based tracking of dependencies, Nix allows for—and in fact enforces—a more precise specification of software versions and computational environments than is common in either package managers or containers. We have nonetheless toned down the statements in the manuscript.

> In the comparison with Guix, the manuscript mixes a statement that I
> would consider neutral (functional vs. data structures) with something
> negative (no types). Maybe try to separate the two, such that
> functional vs DS does not inherit a negative connotation.

We have rewritten the section to remove the negative connotation.

> The conclusion, well, draws the conclusion that BioNix workflows are
> easier to specify, share execute, modify and reproduce. This is a very
> general statement that I do not believe given the arguments in the
> manuscript before. Why is the functional language of Nix easier to
> modify than e.g. a Nextflow or WDL-based workflow definition? In
> particular why is it an advantage to integrate everything into one
> language? For example, speaking about modification: if one just wants
> to modify a software version or a configuration parameter, all one has
> to do with a Snakemake workflow is to edit two YAML files (the
> respective Conda software environment, and the config file). This
> seems easier than with BioNix at first sight.

We have rewritten the conclusion to emphasize 1. the simplicity that comes with using a single platform/language/abstraction to manage environments, software, and workflows, and 2. the strong reproducibility guarantees provided by Nix and BioNix.

> Example 5 compares BioNix with Nextflow and WDL. The caption says that
> the Nextflow pipeline does not capture the software used. I am sorry
> to say this, but such a statement almost upsets me. Nextflow is very
> well able to define this. By adding a single line to each step, one
> could either define a container or an isolated conda environment. I
> suggest to carefully read the documentation of tools before writing
> such comparisons.

We did not intend to convey that Nextflow cannot specify container images nor Conda environments, meerly that the particular example presented does not. This is notable because it is not possible in BioNix: a build that does not completely specify all required software will never execute successfully. The caption has been revised to clarify this and we apologise for the misleading phrasing.

> # Minor comments

> Various paragraphs on page 3 are somehow messed up (incomplete
> sentences). Please carefully check them.

> Page 4, right column, first paragraph: scheduler, not schedulerS.

We have corrected this typo.

**Additional Information:**	
**Question**	**Response**
Are you submitting this manuscript to a special series or article collection?	No

Powered by Editorial Manager® and ProduXion Manager® from Aries Systems Corporation

**Experimental design and statistics**<br><br>Full details of the experimental design and statistical methods used should be given in the Methods section, as detailed in our Minimum Standards Reporting Checklist. Information essential to interpreting the data presented should be made available in the figure legends.<br><br>Have you included all the information requested in your manuscript?	Yes
**Resources**<br><br>A description of all resources used, including antibodies, cell lines, animals and software tools, with enough information to allow them to be uniquely identified, should be included in the Methods section. Authors are strongly encouraged to cite Research Resource Identifiers (RRIDs) for antibodies, model organisms and tools, where possible.<br><br>Have you included the information requested as detailed in our Minimum Standards Reporting Checklist?	Yes
**Availability of data and materials**<br><br>All datasets and code on which the conclusions of the paper rely must be either included in your submission or deposited in publicly available repositories (where available and ethically appropriate), referencing such data using a unique identifier in the references and in the "Availability of Data and Materials" section of your manuscript.<br><br>Have you have met the above requirement as detailed in our Minimum Standards Reporting Checklist?	Yes

Manuscript

Click here to access/download;Manuscript;paper.pdf

Placeholder for OUP logo oup.pdf

GigaScience, 2017, 1–11

doi: xx.xxxx/xxxx
Manuscript in Preparation
Technical Note

TECHNICAL NOTE

Unifying package managers, workflow engines, and containers: computational reproducibility with BioNix

Justin Bedő[1,3,*], Leon Di Stefano[1] and Anthony T. Papenfuss[1,2,4,5,6]

[1]Bioinformatics Division, Walter and Eliza Hall Institute of Medical Research, Parkville, 3052, Victoria, Australia and [2]Peter MacCallum Cancer Centre, Melbourne, VIC 3000, Australia and [3]Department of Computing and Information Systems, University of Melbourne, Melbourne, VIC 3010, Australia and [4]Department of Medical Biology, University of Melbourne, Melbourne, VIC 3010, Australia and [5]Sir Peter MacCallum Department of Oncology, University of Melbourne, Melbourne, VIC 3010, Australia and [6]School of Mathematics and Statistics, University of Melbourne, Melbourne, VIC 3010, Australia

[*] Correspondence author: Justin Bedő, Bioinformatics Division, Walter and Eliza Hall Institute of Medical Research, 3010. Email: bedo.j@wehi.edu.au

Abstract

Motivation: A challenge for computational biologists is to make our analyses reproducible – that is, easy to rerun, combine, and share, with the assurance that equivalent runs will generate identical results. Current best practice aims at this using a combination of package managers, workflow engines, and containers.
Results: We present *BioNix*, a lightweight library built on the Nix deployment system. BioNix manages software dependencies, computational environments, and workflow stages together using a single abstraction: pure functions. This lets users specify workflows in a clean, uniform way, with strong reproducibility guarantees.
Availability and implementation: BioNix is implemented in the Nix expression language and is released on GitHub under the 3-clause BSD license:
`https://github.com/PapenfussLab/bionix`
(biotools:BioNix;RRID:SCR_017662).

Introduction

There are many aspects to the ongoing reproducibility crisis in science – imprecise laboratory protocols, selective reporting, poor use of statistical methods [1, 2] – but for researchers in bioinformatics the most important of these is *computational reproducibility*. Three main challenges exist in practice:

i. *Managing software versions and dependencies*. This is commonly handled with *package managers* (e.g., Conda [3]), which provide both a central repository of software and tools to manage installation on a user's system. Extra repositories for software such as BioConda [4] exist for providing domain specific software.
ii. *Managing computational environments*. This is commonly handled with *containers* (e.g., Docker [5], Singularity [6]) or *virtual machines*; these provide controlled environments within which workflows can be executed.
iii. *Managing workflows*. This is commonly handled with *workflow engines* (e.g., Toil [7], NextFlow [8]), which manage *stages*[1] and their execution, providing features like parallelism, remote building, resumability, and logging.

All of these challenges need to be addressed *at scale*: bioinformatics workflows are computationally demanding, and often need to be executed on computing clusters, on remote computing farms, or in the cloud.

The combination of technologies used to address these issues are called a *reproducibility stack* by Grüning, Chilton, Köster, et al. [9].

Our contributions. We present BioNix, a lightweight library that cleanly deals with all three of these challenges within the one system.

Two distinctive aspects of BioNix's design enable these improvements. The first is that BioNix is built on *Nix*, a next generation cross-platform software deployment system. The second is that in BioNix, stages of a workflow are modelled as *pure functions* – i.e., functions that are free of side effects: workflow stages cannot modify shared state, and so are extremely modular.

These design choices give BioNix several novel features,

1 We define a *stage* as the concrete execution of one or more executables on one or more input files, producing one or more output files.

which we explain using the complete workflow and associated build graph depicted in Example 1:

i. BioNix *manages both software and workflows within the one system*. The build graph in Example 1 has nodes corresponding not just to workflow stages and inputs, but also to software dependencies.
ii. Each stage of a BioNix workflow *implicitly specifies its entire computational environment*. Dependencies are tracked down to the kernel level, and each stage is executed in its own sandbox, resulting in strong reproducibility guarantees and obviating the need for containers[2]. In Nix, sandboxing is enabled by default and may be explicitly disabled for either any specific build or globally.
iii. Nix tracks the entire tree of runtime and build time dependencies with *fine grained versioning*. In the example pipeline this means that not only the version of `bwa`, but also the specific versions of `gcc` and `bash` under which `bwa` was compiled, are captured. All of these versions are fixed by specifying which versions of BioNix and `Nixpkgs` we use (by their commit hashes): the code on the right forms a fully reproducible specification of the associated workflow.
At the same time, it is straightforward to specify specific versions of software for distinct stages, and to use distinct versions of a given piece of software in parallel.
iv. BioNix uses *a simple, purely functional domain-specific language* – the Nix expression language – for specifying workflows. Constructing a workflow is reduced to function composition; stages, workflows, and software dependencies are all represented as pure functions from dependencies to outputs. Because of purity, stages are guaranteed not to influence each other except through their inputs and outputs, and so can be safely recombined. An example of this in Example 1 is our ability to compose workflow steps using the higher-order `map` and `pipe` functions.
The Nix expression language can be considered a compromise between the safety of static configuration files and the expressiveness of a general purpose programming language. Configuration files are predictable, but writing them can involve a lot of boilerplate and repetition. General purpose programming languages allow one to abstract away much of this verbosity, but at the cost of some safety and predictability, for example when they allow workflows to modify unrelated parts of the filesystem. Domain-specific languages like Nix aim to be sufficiently expressive without the error-prone power of a general purpose programming language. BioNix is less flexible than workflow managers based on general purpose languages, but comes with stronger reproducibility guarantees.

BioNix includes many features found among the most powerful existing workflow managers. Intermediate files do not need to be named or managed. Multiple versions of the same piece of software can be used simultanously. BioNix workflows are automatically parallelisable, can be executed in High Performance Computing (HPC) environments or in the cloud, and are fully resumable in cases of interrupted execution. BioNix also allows for conditional execution: that is, different stages may be executed depending on previous stages' outputs.

BioNix includes the following components over and above base Nix:

i. A framework for specifying workflows in the Nix expression language.
ii. A library containing some commonly used bioinformatics tools and helpful workflow specification utilities.
iii. A module allowing workflows to be executed on HPC clusters.
iv. Basic typing to capture metadata and prevent invalid workflow specifications.

The rest of the paper first explains the basics of the Nix system and associated expression language. Next, we describe the design and implementation of BioNix. Finally, we describe an example workflow, and compare Bionix with existing bioinformatics workflow managers.

Preliminaries

The Nix deployment system

The Nix deployment system emerged from the work of Dolstra [10] and Dolstra, Jonge, and Visser [11]. Nix was originally designed as a software package manager, but has since been adapted to managing OS configurations (the `NixOS` project [12, 13]). BioNix represents a further extension of Nix to manage bioinformatics workflows.

The Nix system has three main components:

i. *Build products* or *outputs* may be any kind of directory, file, or collection of files. When using Nix as a traditional package manager, the build products typically consist of the compiled binaries and libraries associated with an application. In our case, build products are any output associated with a bioinformatics workflow or stage.
ii. *Derivations* are static configuration files (ending in `.drv`) that specify all of the inputs and procedures required to produce a given build product. If a build product has prerequisites, then its derivation will refer to the derivations corresponding to those prerequisites.
iii. *Nix expressions* are written in a simple, high-level domain-specific language designed for specifying and manipulating derivations. Derivations are represented in the Nix language as collections of name-value pairs – similar to JSON objects – called "sets." Nix expressions may also make use of various built-in design patterns to provide further extensibility and flexibility.

The basic build process in Nix is as follows: a Nix expression is *instantiated* to yield a tree of *derivations* describing how to generate the associated *build products*. Derivations are then *realised* by the build system to produce the build products themselves. Nix expressions, derivations, and build products are somewhat analogous to source code, object files, and compiled binaries.

The Nix expression corresponding to a given build product will generally take the form of a pure function from dependencies to the corresponding output derivation. Using ML-style notation for types, one can represent this as

$$\text{Dependencies} \rightarrow \text{Output}.$$

Nix ensures that derivations are precisely specified by giving both derivations and build products hash-based names. The hash of a derivation is a function of all of the steps required to produce the associated build product, as well as the hashes of all of its dependencies.

The *Nix store*, usually located on the filesystem at `/nix`, provides a single, flat namespace for all derivations and build products and is writable by only the Nix system. Users typically access the store through *environments*: organised collections of

2 Containers and static binaries can still be used in a BioNix workflow if required, but they are generally avoided to reduce side effects.

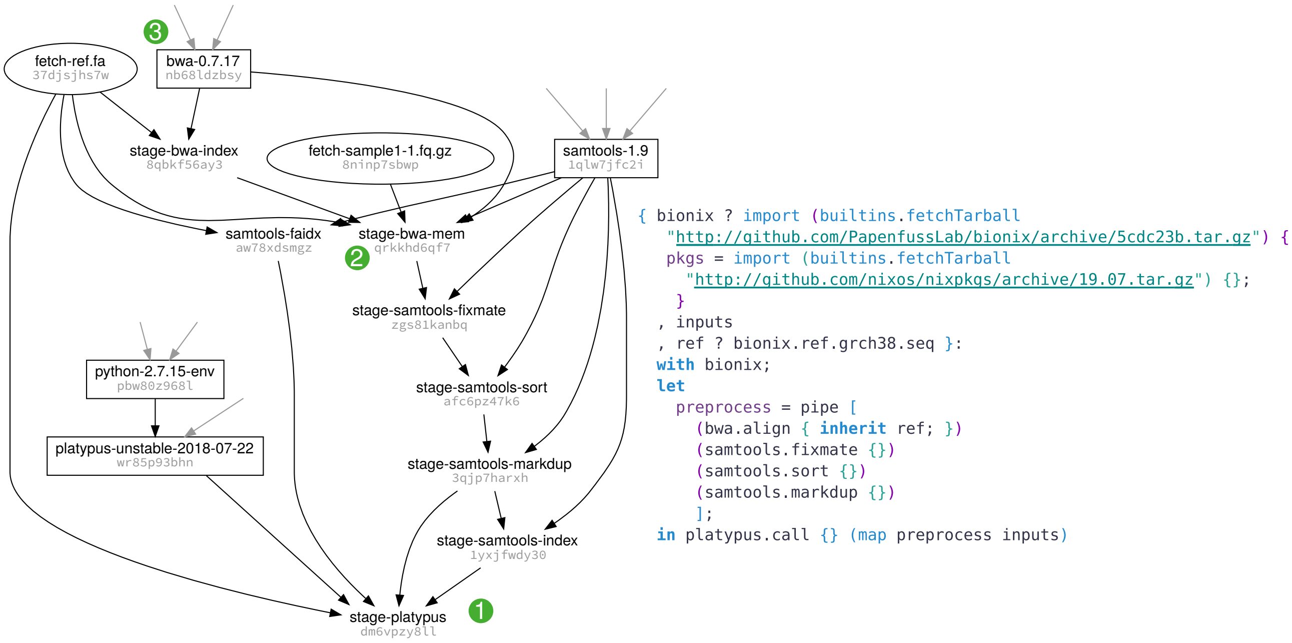


```
{ bionix ? import (builtins.fetchTarball
    "http://github.com/PapenfussLab/bionix/archive/5cdc23b.tar.gz") {
    pkgs = import (builtins.fetchTarball
      "http://github.com/nixos/nixpkgs/archive/19.07.tar.gz") {};
    }
, inputs
, ref ? bionix.ref.grch38.seq }:
with bionix;
let
  preprocess = pipe [
    (bwa.align { inherit ref; })
    (samtools.fixmate {})
    (samtools.sort {})
    (samtools.markdup {})
    ];
in platypus.call {} (map preprocess inputs)
```

Example 1. An example workflow specified in BioNix (right) with a portion of the resulting build graph (left). In the build graph, rectangular nodes correspond to software dependencies and elliptical nodes to data files. Grey arrows indicate dependencies that are not illustrated in the figure. The workflow on the right corresponds to the terminal node in the build graph annotated with (1). The node annotated (2) in the graph corresponds to a single stage in workflow and the corresponding BioNix code can be found in Example 4. The final node annotated (3) corresponds to a software dependency provided by `Nixpkgs` with the corresponding code in Example 6.

soft links exported to `$PATH`.

The Nix community maintains online repository of prebuilt software called `Nixpkgs` [14], which contains over 40,000 software packages.

The Nix expression language

We briefly introduce those parts of the Nix expression language required to understand the rest of the paper.

Sets are the most important datatype in Nix, and correspond to what are sometimes called associative arrays, records, or dictionaries in other languages. Set elements can be accessed by name: `{ a=1; }.a == 1`.

Lists are delimited by square brackets and may contain elements of heterogeneous types separated by whitespace – for example, `[ 1 2 3 ''a'' ''b'' ''c'' true false ]`.

The Nix language makes heavy use of *anonymous functions* (also called *lambda expressions*). The following denotes a function that increments its argument: `x: x + 1`. Nix does not support functions of multiple arguments; instead, it is common for functions to take a set as input. This is written `{ a, b, c, ...}: ...`. Nix allows defaults to be provided for some elements, which are used if the function is called without providing the element. This is denoted using a question mark: the function `{ a ? 5 }: ...` will by default assign `a` the value `5`. Alternatively, one can mimic multi-argument functions using *higher-order* functions – i.e., functions which return functions. For example, `x: y: x + y` denotes a function that adds its two arguments together.

Values can be bound to variable names using the `let ... in ...` construction. We could bind the example above to a name and then invoke it on some parameters: `let f = x: y: x + y; in f 1 2`. Function application is denoted with whitespace (with lower precedence than list elements) and associates to the left; for example `a b c` denotes `(a(b))(c)`. *Pattern matching* allows simultaneous binding of elements contained in a set: `let {a, b} = {a = 1; b = 2;}; in a + b == 3`. Finally, the `with x; ...` construction brings the field names of a set `x` into scope in the subsequent expression.

Implementation

The BioNix library itself is designed as a tree of functions, with each function representing one *stage* of processing. The BioNix tree follows the pattern of `Nixpkgs`; bioinformatics software (e.g., `bwa`, `samtools`, etc) forms the top level, and stages based on subcommands form the second level (e.g., `bwa.align`). As in `Nixpkgs`, defaults can be overridden throughout the whole tree easily.

We will step through three examples of (slightly simplified) BioNix code that generates the build graph in Example 1: the workflow specification, a stage specification, and an expression for a software dependency.

Specifying a workflow

Example 1 shows a simple variant calling workflow using BWA [15, 16] for alignment, samtools [17] for sorting and duplicate marking, and platypus [18] for variant calling. The whole workflow is a single anonymous function, taking dependencies and inputs – the set spanning the first 8 lines – to an output (the final line):

$$\text{(Inputs, Options, \& Dependencies)} \rightarrow \text{Output}.$$

The output of this workflow is the output of platypus, which is a `.vcf` file.

One of the dependencies of the workflow is BioNix itself. If the user does not specify a version to use, the workflow defaults to using the specific commit indicated. Similarly, if the user

does not specify a reference, the workflow defaults to GRCh38. Fixing a version of BioNix and Nixpkgs automatically fixes versions of all software used in the pipline, though these can be individually specifically altered if desired (see Example 2 and Example 3).

```
let
  oldnix = import (fetchFromGitHub {
    owner = "NixOS";
    repo = "nixpkgs";
    rev = "83a893c38a83877588e3ca7ccfeabaa973c30acd";
    sha256 = "0q7214hag7h95irvhkdb648m09b9jspb0raw1qjrx7y4grzb165h";

  }) { };

  jre = oldnix.openjdk7;

in ...
```

Example 2. This example is an extract from the MuTect stage and demonstrates how specific software versions can be referenced. Here the deprecated JDK 7 required by MuTect is accessed through an old revision of Nixpkgs.

Each stage – for example, `bwa.align`, `samtools.fixmate`, or `platypus.call` – is represented by a higher-order function that takes options and dependencies, and returns a function from inputs to outputs. The type of a stage[3] can be represented as

$$(\text{Options \& Dependencies}) \rightarrow (\text{Inputs} \rightarrow \text{Output}).$$

```
let
  octopus-git = octopus-caller.overrideAttrs (attrs: {
    src = fetchFromGitHub {
      owner = "luntergroup";
      repo = "octopus";
      rev = "f88d35b9b93d11a086eb87bb3722874a3ea5250e";
      sha256 = "171cmpx6x8p1q4d0k9lj2mwcyhhrc20cskqhwdyl6fa1clx40b7r";
    };
  });
in ...
```

Example 3. Nixpkgs has a flexible overrides system that allows derivations to be selectively modified. Here the Octopus variant caller switched to the latest development branch (as of 2020-03-20) instead of the current release.

BioNix dependencies by default include the BioNix tree itself – to allow use of other (sub-)stages – and well as the `Nixpkgs` collection, which provides the necessary general-purose software. For most of our stages we do not pass in any additional options, and so the first argument is `{}`. However, `bwa.align` requires that we specify a reference, and so we explicitly pass in the `ref` declared at the beginning of the workflow.

We make use of several helpful abstractions from functional programming. For example, we define a new function, `preprocess`, that takes a sample and performs alignment, mate-fixing, sorting, and duplicate-marking. We also use the `pipe` function in BioNix to sequentially compose a list of functions. Finally, we `map` this function over all our inputs. The Nix expression language allows for this abstraction and modularity without introducing side-effects.

```
{ bionix
, ref
, bamOutput ? true
}:

{ input1
, input2 ? null
}:

with bionix;
with lib;

stage {
  name = "bwa-mem";
  buildInputs = with pkgs; [ bwa ] ++
    optional bamOutput samtools;
  buildCommand = ''
    ln -s ${ref} ref.fa
    for f in ${bionix.bwa.index {} ref}/* ; do
      ln -s $f
    done

    bwa mem -t $NIX_BUILD_CORES \
      ref.fa \
      ${fq input1} \
      ${optionalString (input2 != null) input2} \
      ${optionalString bamOutput "| samtools view -b"} \
      > $out
  '';
}
```

Example 4. Specifying an alignment stage using BWA-mem. The expression defines a function mapping parameters (e.g., a choice of reference genome) and the fastq inputs to a derivation produced by the `stage` function. The `stage` function takes as arguments a build script and the requisite software.

Specifying a stage

Example 4 illustrates an example *stage* in BioNix. In line with our design pattern, the whole stage is represented by an anonymous higher-order function: it takes a record of options and dependencies, and returns a function that takes inputs – in this case, a pair of FASTQ files representing read pairs – and returns a derivation. Notice that we give the reference as part of the first argument to the stage (options and dependencies) rather than as part of the second argument (inputs). This is because often an entire workflow will be parametrised by a single reference genome.

Links are created for both the reference and its associated BWA indices to deal with the standard bioinformatics convention that indices are located in the same directory as the associated indexed file.

Finally, the output is optionally converted to the `.bam` format within the shell script associated with the derivation. BioNix cannot stream data between stages of a workflow: both inputs and outputs of a stage must be a file or set of files.

Example 4 has multiple inputs and only a single output (the BAM file), however multiple outputs are also supported by Nix derivations. Example 5 demonstrates multiple outputs for picard tools [19] where metrics in addition to the main output. The extra output can be accessed via the metrics attribute in the returned derivation.

3 Functional programmers will recognise this as a *curried* version of the type of a workflow.

```
stage {
    name = "picard-markDuplicates";
    buildInputs = with pkgs;
        [ picard-tools ];
    outputs = [ "out" "metrics" ];
    buildCommand = ''
        picard MarkDuplicates \
            I=${inputBam} \
            O=$out \
            M=$metrics
        '';
}
```

Example 5. Extract from the definition of the mark duplicates expression for picard tools demonstrating multiple outputs. The output attribute names the build products, which are assigned unique paths in the store and exposed to the build script via environment variables of the same name.

Specifying a software dependency

For completeness, we also show how to specify a software dependency. In our example workflow, the BWA software is provided by `Nixpkgs` and Example 6 shows a simplified version of its specification there.

The expression is an anonymous function from dependencies – in this case, the utility libraries `stdenv` and `fetchurl` and the C library dependency `zlib` – to outputs – in this case, the compiled binary for `bwa`. The function body is just a single call to the helper function `mkDerivation`. Since `bwa` follows the first two parts of the common `./configure; make; make install` pattern for building unix software, only the final install phase needs to be specified. Here, the resulting binary is copied into the `bin/` directory.

```
{ stdenv, fetchurl, zlib } :

stdenv.mkDerivation rec {
  name = "bwa-${version}";
  version = "0.7.17";

  src = fetchurl {
    url = "mirror://sourceforge/bio-bwa/${name}.tar.bz2";
    sha256 = "1zfhv2zg9v1icdlq4p9ssc8k01mca5d1bd87w71py2swfi74s6yy";
  };

  buildInputs = [ zlib ];

  installPhase = ''
    install -vD bwa $out/bin/bwa
  '';
}
```

Example 6. Specifying a software dependency for bwa-mem. This is a simplified version of the expression found in `Nixpkgs`. The expression defines the build requirements (zlib) and the steps required to build the software. A standard build process (`configure`, `make`, `make install`) is assumed, so only non-standard commands need to be specified. BWA does not support the standard `make install` for installation, so an install script is defined in the expression.

HPC queue integration

While the Nix build system provides support for both local and remote building, bioinformatics workflows are commonly executed on traditional HPC infrastructure managed by a job schedulers. These systems require users to submit jobs to a queue, along with specified resource limits.

BioNix provides support for queuing systems via a function that takes resource limits and a derivation, and returns a new derivation that will submit the build process as a job to the queuing system instead of building it directly. This design allows arbitrary derivations to be lifted to the queue, and also allows users to combine submission to the queue and building via the Nix build system directly. However, as submission is a (relatively benign) side effect, builds cannot be realised using sandboxing. This is because the default sandbox prevents the build from using software not specified in the expression, and submitting jobs to the scheduler requires interacting with the daemon running outside the build environment. This restriction only applies to cluster execution; local and remote builds fully support sandboxing.

Failures in the queue are handled similarly to execution failures: the build is aborted and reported to the user. This includes when jobs are terminated due to resource limits. The jobid of the submission is recorded in the build log along with any output produced by the job to aid the user in tracing the error.

Tracking types of build products

BioNix gives build products optional *types* in order to prevent errors in workflow specification and to track useful metadata such as the reference used for an alignment. This is a lightweight version of the approach taken by *Bioshake* [20, 21]. Types are implemented as an abstract data type (ADT) and are tracked using Nix's passthru features.

Discussion

Real world use of BioNix

Small variant calling workflow. We have used BioNix to manage a workflow that performs somatic variant calling and Copy Number Variant (CNV) calling on whole genome deep sequencing human data using Minimap2 [22] for alignment, samtools [17] for sorting and marking duplicates, Strelka [23] for somatic variant calling, and CNVkit [24] for CNV calling.

This workflow was executed on HPC infrastructure managed with the TORQUE resource manager [25] using the extensions presented earlier. A total of 1.1TB of (compressed) fastq input was processed, producing 755GB of results (including alignments). The workflow is detailed in Example 7 and a full example executing the workflow on a publicly available melanoma dataset [26] is available in the BioNix repository.

Structural variant calling at scale. We have also used BioNix to execute a workflow that processes 6.8TB of whole genome sequencing data from mice, performing quality checking, alignment, and merging, and structural variant calling using `gridss` [27] with a range of parameters. This resulted in a total of 5.3TB of results. See Example 8 for the workflow used.

Limitations of BioNix

BioNix leverages the underlying Nix system to achieve its reproducibility, and consequently is subject to the same limitations present in Nix.

A given stage may only write to the store location assigned to it, so streaming data between two distinct stages is not possible. Streaming steps must therefore be combined into one stage, which can be constructed with higher order functions. Streaming between two independent builds would be difficult

```
{bionix ? import <bionix> {}, pair, fetch}:

with bionix;
with lib;
with types;

with minimap2;
with samtools;
with snpeff;

let
  preprocess = s: pipe s [
    fetch
    (align { preset = "sr"; ref = ref.grch38.seq; flags = "-R'@RG\\tID:${s.type}\\tSM:${s.type}'"; })
    (fixmate {})
    (sort { })
    (markdup { })
  ];

  dropErrors = input: stage {
    name = "drop-errors";
    buildCommand = ''
      grep -v "ERROR_" ${input} > $out
    '';
    passthru.filetype = input.filetype;
  };

  bams = mapAttrs (_: preprocess) pair;

  variants = let
    somatic = strelka.callSomatic { } bams; in mapAttrs (_: flip pipe [
      (compression.uncompress { })
      (snpeff.annotate { db = ref.grch38.snpeff.db; })
      dropErrors
      (snpeff.dbnsfp { dbnsfp = ref.grch38.snpeff.dbnsfp; })
    ]) {
      "snvs.vcf" = somatic.snvs;
      "indels.vcf" = somatic.snvs;
      "germline.vcf" = strelka.call { } [bams.normal];
    };

  cnvs = cnvkit.callCNV { } { normals = [ bams.normal ]; tumours = [ bams.tumour ]; };

in linkOutputs {
  inherit variants;
  alignments = linkOutputs (mapAttrs' (n: nameValuePair (n + ".bam")) bams);
  cnvkit = cnvs;
}
```

Example 7. The tumour-normal small variant calling workflow used for calling variants on clinical samples. Reads are aligned using Minimap2 [22], variants called using Strelka [23], and finally CNVs with CNVkit [24]. The inputs are a pair of samples (as an attribute set containing `normal` and `tumour` attributes), a method `fetch` for fetching the reads associated with a given sample, and BioNix.

```
{ bionix, baseUrl, mice ? import ../metadata/mice.nix }:

with bionix;
with lib;

let
  # Utility function
  update = f: x: x // (f x);

  # Process fastqs ############################################
  fetch = { filename, sha256sum, ... }:
    fetchFastQGZ {
      url = baseUrl + filename;
      sha256 = sha256sum;
    };

  updateFastq = update (fq: { fastqcOutput = fastqc.check { } (fetch fq); });

  # Process sample run ########################################
  fetchInputs = { fq1, fq2, ... }: {
    input1 = fetch fq1;
    input2 = fetch fq2;
  };

  alignSortSampleRun = sr:
    pipe sr [
      fetchInputs
      (bwa.align {
        ref = ref.grcm38.seq;
        flags = "-R'@RG\\tID:${sr.id_col}\\tSM:${sr.sample_id}'";
      })
      (samtools.sort { })
    ];

  updateSampleRun = update (sr: {
    sampleRunBam = alignSortSampleRun sr;
    # recurse
    fq1 = updateFastq sr.fq1;
    fq2 = updateFastq sr.fq2;
  });

  # Process samples ###########################################
  mergedBam = flip pipe [ (map (sr: sr.sampleRunBam)) (samtools.merge { }) ];

  updateSample = update (sample:
    let updated_sample_runs = map updateSampleRun sample.sample_runs;
    in {
      sample_runs = updated_sample_runs;
      mergedBam = mergedBam updated_sample_runs;
    });

  # Process mice ##############################################
  updateMouse = update (mouse: rec {
    gridssCalls =
      gridss.callAndAssemble (map (sample: sample.mergedBam) samples);
    samples = map updateSample mouse.samples;
  });

in map updateMouse mice
```

Example 8. Structural variant calling for a mouse dataset. Stages include quality checking with FastQC [28], alignment with BWA [15], merging with samtools [17] and finally structural variant calling with GRIDSS [27]. The inputs to the expression are BioNix, a base URL where the fastq files can be found, and the metadata describing the experimental design, sequencing data and hashes. This workflow is structured so that metadata are "annotated" with build products, analogous to building up a data structure in a general-purpose language. Since Nix is lazy, the build products will only be built when requested. Comparing with 7 shows how flexibly workflows can be specified within BioNix.

to support: the distributed design of Nix implies that different builds may be executing on independent machines.

Nix and BioNix will currently rebuild an output unnecessarily when dependencies of its inputs have changed, but the inputs themselves have not. The reason is that the Nix store is not content addressed; store locations are based on the cryptographic hash of all inputs used in building an output, rather than the output itself. This has been referred to as an *extensional* model [11]. The proposed *intensional* store model [11] introduces content addressable storage and hash rewriting, allowing better sharing of components and reducing unnecessary builds. This feature is currently under implementation and is not available in the latest Nix release (2.3.1).

The Nix language, though extremely simple, has an idiosyncratic syntax that draws from both curly brace and functional programming languages; some may find this unfamiliar or off-putting.

Finally, Nix does not have an advanced type system. BioNix provides type safety for many of its stages through an implementation of ADTs, but as these data types are implemented in Nix itself the error reporting can be obscure.

Related work

We discuss here two categories of work related to our own. The first consists of other projects making use of the Nix deployment system to manage data processing workflows; the second concerns existing workflow management tools popular in bioinformatics and computational biology.

Similar adaptations of the Nix system

Several groups have made use of Nix to manage the *environments* in which computational workflows are executed. Researchers at GRICAD at the Université Grenoble Alpes have made use of Nix as an HPC package management system [29, 30]. The Pipelines in Genomics (PiGx) project [31] uses Guix – an implementation of the Nix system using GNU Scheme in place of the Nix expression language – to produce a set of reproducible "turn-key" workflows for bioinformatics and computational biology, configured via simple static config files. Similar uses of Nix for reproducible research have also been suggested by Blair Archibald of the Software Sustainability Institute [32, 33] and Bruno Vieira at the Mozilla Foundation [34].

However, none of these approaches use Nix to specify workflows themselves; instead, Nix is used as a replacement for package managers and containers. BioNix takes the next step and embeds the workflows into the Nix system.

Two projects that we know of make use of Nix to manage workflows themselves: Mix, a Nix-based system for specifying data processing pipelines developed at SoundCloud [35], and Fractalide, a service programming platform using dataflow graphs [36].

Mix is built on the `hnix` project [37] and implements a new builder dedicated to data workflows. Mix redefines derivations to remove the Nix store and allow storage of products on a distributed file system. Consequently, Mix cannot take advantage of `Nixpkgs` and focuses entirely on the workflows, without capturing the associated computational environments.

Fractalide is an effort to provide a dataflow graph programming platform with an initial focus on microservices and the internet of things. Though it builds on Nix, it also extends the base language with a new language for specifying the dataflow graphs, and relies on bindings to other languages to provide an interface to the actual data processing (i.e., the microservice). By contrast, BioNix focuses on Bioinformatics workflows, is implemented entirely within the existing Nix ecosystem, and calls existing pieces of software via their command line interfaces.

The Guix Workflow Language (GWL) [38, 39] is in many ways the workflow manager closest in approach to BioNix. GWL, like PiGx, is built on Guix, and so inherits the reproducibility guarantees of a Nix-like system. Unlike PiGx, GWL manages workflows themselves using Guix, rather than using it only to provide the necessary software environment. However unlike BioNix, stages in GWL are not represented by functions but by data structures; workflows are specified via manual construction of the associated build graph; and workflow stages are untyped.

Existing workflow managers for computational biology

As already mentioned, current best practice aims at reproducibility using a combination of package managers, containers, and workflow engines. BioNix combines the functionality of all of these, and in this sense is difficult to compare with existing workflow management tools.

However, we can compare the syntax of BioNix with that of existing workflow managers by implementing toy piplines in each. Examples 9 and 10 illustrate two simple examples from the documentation of Workflow Description Language (WDL) [40] and NextFlow [8] alongside the equivalent BioNix expression. BioNix necessarily defines the software used in the execution of the workflow, and software outside of Nix is unavailable. By contrast, the WDL and NextFlow examples presented are valid workflow specifications without software definitions.

BioNix might also be compared with Common Workflow Language (CWL) [43], which is a standard specification language intended for describing workflows in a portable way. However, CWL is increasingly used as a target for other build systems, rather than being written directly. In this sense, CWL increasingly plays a role similar to Nix's derivation files, which are complete, portable, machine-readable specifications that can be built on local or remote systems.

Galaxy [44] is a popular workflow platform that provides a web-based GUI for specification of workflows and execution controls. Galaxy provides facilities to manage the computational environment via various package management tools, with Conda being popular. Nix can be integrated into Galaxy, which would allow Galaxy to leverage the strong reproducibility guarantees of Nix [10, 11]. This would be similar to the approach taken by PiGx.

Cuneiform [45, 46] is a functional programming language for large-scale data analysis workflows. In Cuneiform, as in BioNix, workflow stages are modelled as pure functions. Cuneiform also has an elegant foreign function interface (FFI), allowing the seamless use of code snippets from a variety of languages – bash, Python, R, and others – as well as a language-level static type system. On the other hand, Cuneiform does not manage software dependencies, and so lacks the reproducibility guarantees that BioNix leverages from Nix.

Finally, SciPipe [47] is a recent workflow library that focuses on dynamic execution and streaming. Like Bionix, SciPipe provides logs at the resolution of each build and allows incremental (partial) builds. On the other hand, SciPipe has a strong focus on streaming which is not supported in Nix between independent builds (see).

Conclusions

We have presented BioNix, a framework built on Nix in which workflows are specified using pure functions. BioNix captures software versions and dependencies, manages computational environments, and composes the various stages of workflows all within the one framework and language. Previous ap-

```nix
{ bionix ? import <bionix> {} }:

with bionix;
with lib;

let

  prepare = splitString "\n" (removeSuffix "\n" (readFile (stage {
    name = "prepare";
    buildInputs = [ pkgs.python3 ];
    buildCommand = ''
      python -c "print('one\ntwo\nthree\nfour', end=''')" > $out
    '';
  })));

  analysis = str: removeSuffix "\n" (readFile (stage {
    name = "analysis";
    buildInputs = [ pkgs.python ];
    buildCommand = ''
      python -c "print('_${str}_')" > $out
    '';
  }));

  gather = strs: stage {
    name = "gather";
    buildCommand = ''
      echo ${concatStringsSep " " strs} > $out
    '';
  };

in gather (map analysis prepare)
```

```wdl
task prepare {
  command <<<
    python -c "print('one\ntwo\nthree\nfour')"
  >>>
  output {
    Array[String] array = read_lines(stdout())
  }
}

task analysis {
  String str
  command <<<
    python -c "print('_${str}_')"
  >>>
  output {
    String out = read_string(stdout())
  }
}

task gather {
  Array[String] array
  command <<<
    echo ${sep=' ' array}
  >>>
  output {
    String str = read_string(stdout())
  }
}

workflow example {
  call prepare
  scatter (x in prepare.array) {
    call analysis {input: str=x}
  }
  call gather {input: array=analysis.out}
}
```

Example 9. Scatter-gather example from WDL [41] documentation with the BioNix implementation on the left and WDL on the right. The workflow generates some input data using python, parses it into lines, transforms each line via a simple python script, then collects all lines into a final output. It is unusual to parse and split using the Nix language – typically this would instead be done through a build – but we have done so to maintain a closer translation of the WDL example. In BioNix we must specify the python dependency: as the entire software environment is managed, a failure to specify software will result in a failed build. The BioNix example also shows how different software versions can be combined: Python 3 is used in the `prepare` stage, but Python 2 is used in the `analysis` stage.

```nix
{ bionix ? import <bionix> {}
, input ? ./sample.fa}:

with bionix;
with lib;

let
  splitSequences = fa: stage {
    name = "splitSequences";
    buildInputs = [ pkgs.gawk ];
    buildCommand = ''
      awk '/^>/{f="seq_"++d} {print > f}' ${fa}
      mkdir $out
      cp seq* $out
    '';
  };

  reverse = fa: stage {
    name = "reverse";
    buildCommand = ''
      ${pkgs.utillinux}/bin/rev ${fa} > $out
    '';
  };

in pipe [
  splitSequences
  (each reverse)
] input
```

```nextflow
params.in = "$baseDir/data/sample.fa"
sequences = file(params.in)

/*
 * split a fasta file in multiple files
 */
process splitSequences {

    input:
    file 'input.fa' from sequences

    output:
    file 'seq_*' into records

    """
    awk '/^>/{f="seq_"++d} {print > f}' < input.fa
    """

}

/*
 * Simple reverse the sequences
 */
process reverse {

    input:
    file x from records

    output:
    stdout result

    """
    cat $x | rev
    """
}

/*
 * print the channel content
 */
result.subscribe { println it }
```

Example 10. Nextflow basic pipeline example [42] (right) translated to BioNix (left). The example splits a single FastA file into a collection of FastA files, each containing exactly one sequence. The sequences are then reversed (in parallel) and then gathered back into one file in the final step. The BioNix `pipe` function implements reverse function composition for the easy specification of sequences of stages. The BioNix expression requires us to specify which `awk` implementation to use; here we chose GNU Awk.

proaches to computational reproducibility have relied on a combination of technologies such as containers, package managers, and workflow engines to achieve the same ends. BioNix unites these functionalities under the one framework, making it simple to specify computational biology workflows with strong reproducibility guarantees.

BioNix is available at http://github.com/PapenfussLab/bionix under the 3-clause BSD license.

Acknowledgements

Thanks to Ramyar Molania and Jocelyn Sietsma Penington for being early adopters of BioNix, and to Ramyar for helpful comments on the manuscript. Thanks to Alan Rubin for many helpful discussions and comments on the manuscript.

A.T.P. was supported by the Lorenzo and Pamela Galli Charitable Trust and by an Australian National Health and Medical Research Council (NHMRC) Program Grant (1054618) and NHMRC Senior Research Fellowship (1116955). The research benefitted by support from the Victorian State Government Operational Infrastructure Support and Australian Government NHMRC Independent Research Institute Infrastructure Support.

J.B. was supported by the Stafford Fox Medical Research Foundation.

Competing interests

The authors declare that they have no competing interests.

References

1. Reality check on reproducibility. Nature 2016;533:437–7.
2. Challenges in irreproducible research. Nature, 2018.
3. Package, dependency and environment management for any language—Python, R, Ruby, Lua, Scala, Java, JavaScript, C/ C++, FORTRAN. 2018. URL: https://conda.io/docs/.
4. Grüning B, Dale R, Sjödin A, et al. Bioconda: sustainable and comprehensive software distribution for the life sciences. Nature Methods 2018;15:475–6.
5. Enterprise container platform. 2018. URL: https://www.docker.com.
6. Singularity. 2018. URL: https://www.sylabs.io/singularity/.
7. Vivian J, Rao AA, Nothaft FA, et al. Toil enables reproducible, open source, big biomedical data analyses. Nature Biotechnology 2017;35:314–6.
8. Di Tommaso P, Chatzou M, Floden EW, Barja PP, Palumbo E, and Notredame C. Nextflow enables reproducible computational workflows. Nature Biotechnology 2017;35:316–9.
9. Grüning B, Chilton J, Köster J, et al. Practical Computational Reproducibility in the Life Sciences. Cell Systems 2018;6:631–5.
10. Dolstra E. The Purely Functional Software Deployment Model. PhD thesis. Faculty of Science, Utrecht, The Netherlands, 2006.
11. Dolstra E, Jonge M de, and Visser E. Nix: A Safe and Policy-Free System for Software Deployment. In: *Proceedings of the 18th Large Installation System Administration Conference* (Atlanta). 2004.
12. Dolstra E, Löh A, and Pierron N. NixOS: A Purely Functional Linux Distribution. Journal of Functional Programming 2010:577–615.
13. NixOS. 2019. URL: https://www.nixos.org/nixos.
14. nixpkgs. 2019. URL: https://www.nixos.org/nixpkgs.
15. Li H. Aligning sequence reads, clone sequences and assembly contigs with BWA-MEM. 2013. arXiv: q-bio/1303.3997.
16. Li H and Durbin R. Fast and accurate short read alignment with Burrows-Wheeler transform. Bioinformatics 2009;25:1754–60.
17. Li H, Handsaker B, Wysoker A, et al. The Sequence Alignment/Map format and SAMtools. Bioinformatics 2009;25:2078–9.
18. Rimmer A, Phan H, Mathieson I, et al. Integrating mapping-, assembly- and haplotype-based approaches for calling variants in clinical sequencing applications. Nature Genetics 2014;46:912–8.
19. Picard toolkit. http://broadinstitute.github.io/picard/. 2019.
20. Bedő J. Bioshake: a Haskell EDSL for bioinformatics pipelines. 2018. bioRxiv: http://doi.org/10.1101/529479.
21. Bioshake. 2019. URL: https://github.com/PapenfussLab/bioshake.
22. Li H. Minimap2: pairwise alignment for nucleotide sequences. Bioinformatics 2018;34. Ed. by Birol I:3094–100.
23. Kim S, Scheffler K, Halpern AL, et al. Strelka2: fast and accurate calling of germline and somatic variants. Nature Methods 2018;15:591–4.
24. Talevich E, Shain AH, Botton T, and Bastian BC. CNVkit: Genome-Wide Copy Number Detection and Visualization from Targeted DNA Sequencing. PLOS Computational Biology 2016;12:e1004873.
25. TORQUE Resource Manager. 2019. URL: http://www.adaptivecomputing.com/products/torque/.
26. Cameron DL, Baber J, Shale C, et al. GRIDSS, PURPLE, LINX: Unscrambling the tumor genome via integrated analysis of structural variation and copy number. 2019.
27. Cameron DL, Schröder J, Penington JS, et al. GRIDSS: sensitive and specific genomic rearrangement detection using positional de Bruijn graph assembly. Genome Research 2017;27:2050–60.
28. Andrews S, Krueger F, Segonds-Pichon A, Biggins L, Krueger C, and Wingett S. FastQC. Babraham Institute. Babraham, UK, 2010.
29. Bzeznik B, Henriot O, Reis V, Richard O, and Tavard L. Nix as HPC package management system. In: *Proceedings of the Fourth International Workshop on HPC User Support Tools - HUST'17*. the Fourth International Workshop. Denver, CO, USA: ACM Press, 2017:1–6. DOI: 10.1145/3152493.3152556.
30. Bouttier PA. Nix as HPC package management system. NixCon. 2018.
31. Wurmus R, Uyar B, Osberg B, et al. PiGx: reproducible genomics analysis pipelines with GNU Guix. GigaScience 2018;7.
32. Archibald B. Reproducible Environments With Nix. Software Sustainability Institute. 2017. URL: https://www.software.ac.uk/blog/2017-10-05-reproducible-environments-nix (visited on 01/23/2019).

33. Crouch S, Hong NC, Hettrick S, et al. The Software Sustainability Institute: Changing Research Software Attitudes and Practices. Computing in Science Engineering 2013;15:74–80.

34. Vieira B. A truly reproducible scientific paper? Bruno Vieira. 2017. URL: https://medium.com/@bmpvieira/a-truly-reproducible-scientific-paper-5059b282ee9a (visited on 01/23/2019).

35. Georges Dubus. Mix: Nix for data pipeline configuration. NixCon. London, 2018.

36. Reusable Reproducible Composable Software. 2019. URL: https://github.com/fractalide/fractalide.

37. A Haskell re-implementation of the Nix expression language. 2019. URL: https://github.com/haskell-nix/hnix.

38. Janssen, Roel. Workflow management with GNU Guix. FOSDEM 2017. 2017.

39. Wurmus R. GWL: GNU Workflow Language. FOSDEM 2019. 2019.

40. WDL | Home. URL: https://software.broadinstitute.org/wdl/ (visited on 01/23/2019).

41. Workflow Description Language - Specification and Implementations. 2019. URL: https://github.com/openwdl/wdl#scattergather.

42. Nextflow - Basic pipeline. 2019. URL: https://www.nextflow.io/example1.html.

43. Amstutz P, Crusoe MR, Tijanić N, et al. Common Workflow Language, v1.0. 2016. DOI: 10.6084/m9.figshare.3115156.v2.

44. Afgan E, Baker D, Batut B, et al. The Galaxy platform for accessible, reproducible and collaborative biomedical analyses: 2018 update. Nucleic Acids Research 2018;46:W537–W544.

45. Brandt J, Bux M, and Leser U. Cuneiform: A Functional Language for Large Scale Scientific Data Analysis. In: *Proceedings of the Workshops of the EDBT/ICDT*. Vol. 1330. Brussels, Belgium, 2015:17–26.

46. Brandt J, Reisig W, and Leser U. Computation semantics of the functional scientific workflow language Cuneiform*. Journal of Functional Programming 2017;27.

47. Lampa S, Dahlö M, Alvarsson J, and Spjuth O. SciPipe: A workflow library for agile development of complex and dynamic bioinformatics pipelines. GigaScience 2019;8.

Manuscript diff markup

Click here to access/download
Supplementary Material
diff.pdf
