# Supplementary material for: Unifying package managers, workflow engines, and containers: Computational reproducibility with BioNix
Source: Gigascience. 2020 Nov 18;9(11):giaa121. doi: 10.1093/gigascience/giaa121 (PMC7672450; doi:10.1093/gigascience/giaa121)
Supplement: giaa121_GIGA-D-19-00324_Revision_3 [file giaa121_giga-d-19-00324_revision_3.pdf]

GigaScience

Unifying package managers, workflow engines, and containers: computational reproducibility with BioNix

--Manuscript Draft--

Manuscript Number:	GIGA-D-19-00324R3
Full Title:	Unifying package managers, workflow engines, and containers: computational reproducibility with BioNix
Article Type:	Technical Note
Funding Information:	Australian National Health and Medical Research Council (1054618) — Dr Anthony T. Papenfuss<br>Australian National Health and Medical Research Council (1116955) — Dr Anthony T. Papenfuss
Abstract:	Motivation: A challenge for computational biologists is to make our analyses reproducible -- that is, easy to rerun, combine, and share, with the assurance that equivalent runs will generate identical results.<br>Current best practice aims at this using a combination of package managers, workflow engines, and containers.<br><br>Results: We present BioNix, a lightweight library built on the Nix deployment system.<br>BioNix manages software dependencies, computational environments, and workflow stages together using a single abstraction: pure functions.<br>This lets us specify workflows in a way that is more reproducible and modular than current best practices.<br><br>Availability and implementation: BioNix is implemented in the Nix expression language and is released on GitHub under the 3-clause BSD license: https://github.com/PapenfussLab/bionix.
Corresponding Author:	Justin Bedo<br><br>AUSTRALIA
Corresponding Author Secondary Information:	
Corresponding Author's Institution:	
Corresponding Author's Secondary Institution:	
First Author:	Justin Bedo
First Author Secondary Information:	

Powered by Editorial Manager® and ProduXion Manager® from Aries Systems Corporation

**Order of Authors:**	Justin Bedo
	Leon Di Stefano
	Anthony T. Papenfuss
**Order of Authors Secondary Information:**	
**Response to Reviewers:**	GigaDB reference and "Availability of supporting source code and requirements" section added as requested.
**Additional Information:**	
**Question**	**Response**
Are you submitting this manuscript to a special series or article collection?	No
**Experimental design and statistics**<br><br>Full details of the experimental design and statistical methods used should be given in the Methods section, as detailed in our Minimum Standards Reporting Checklist. Information essential to interpreting the data presented should be made available in the figure legends.<br><br>Have you included all the information requested in your manuscript?	Yes
**Resources**<br><br>A description of all resources used, including antibodies, cell lines, animals and software tools, with enough information to allow them to be uniquely identified, should be included in the Methods section. Authors are strongly encouraged to cite Research Resource Identifiers (RRIDs) for antibodies, model organisms and tools, where possible.<br><br>Have you included the information requested as detailed in our Minimum Standards Reporting Checklist?	Yes
**Availability of data and materials**<br><br>All datasets and code on which the conclusions of the paper rely must be either included in your submission or	Yes

deposited in publicly available repositories (where available and ethically appropriate), referencing such data using a unique identifier in the references and in the "Availability of Data and Materials" section of your manuscript.<br><br>Have you have met the above requirement as detailed in our Minimum Standards Reporting Checklist?	

Powered by Editorial Manager® and ProduXion Manager® from Aries Systems Corporation

Manuscript
Click here to access/download;Manuscript;revised paper.pdf

Placeholder for OUP logo oup.pdf

GigaScience, 2017, 1–12

doi: xx.xxxx/xxxx
Manuscript in Preparation
Technical Note

TECHNICAL NOTE

Unifying package managers, workflow engines, and containers: computational reproducibility with BioNix

Justin Bedő[1,3,*], Leon Di Stefano[1] and Anthony T. Papenfuss[1,2,4,5,6]

[1]Bioinformatics Division, Walter and Eliza Hall Institute of Medical Research, Parkville, 3052, Victoria, Australia and [2]Peter MacCallum Cancer Centre, Melbourne, VIC 3000, Australia and [3]Department of Computing and Information Systems, University of Melbourne, Melbourne, VIC 3010, Australia and [4]Department of Medical Biology, University of Melbourne, Melbourne, VIC 3010, Australia and [5]Sir Peter MacCallum Department of Oncology, University of Melbourne, Melbourne, VIC 3010, Australia and [6]School of Mathematics and Statistics, University of Melbourne, Melbourne, VIC 3010, Australia

* Correspondence author: Justin Bedő, Bioinformatics Division, Walter and Eliza Hall Institute of Medical Research, 3010. Email: bedo.j@wehi.edu.au

Abstract

Motivation: A challenge for computational biologists is to make our analyses reproducible – that is, easy to rerun, combine, and share, with the assurance that equivalent runs will generate identical results. Current best practice aims at this using a combination of package managers, workflow engines, and containers.
Results: We present *BioNix*, a lightweight library built on the Nix deployment system. BioNix manages software dependencies, computational environments, and workflow stages together using a single abstraction: pure functions. This lets users specify workflows in a clean, uniform way, with strong reproducibility guarantees.
Availability and implementation: BioNix is implemented in the Nix expression language and is released on GitHub under the 3-clause BSD license:
`https://github.com/PapenfussLab/bionix`
(biotools:BioNix;RRID:SCR_017662). A GigaDB archival snapshot is also available [1].

Introduction

There are many aspects to the ongoing reproducibility crisis in science – imprecise laboratory protocols, selective reporting, poor use of statistical methods [2, 3] – but for researchers in bioinformatics the most important of these is *computational reproducibility*. Three main challenges exist in practice:

i. *Managing software versions and dependencies*. This is commonly handled with *package managers* (e.g., Conda [4]), which provide both a central repository of software and tools to manage installation on a user's system. Extra repositories for software such as BioConda [5] exist for providing domain specific software.
ii. *Managing computational environments*. This is commonly handled with *containers* (e.g., Docker [6], Singularity [7]) or *virtual machines*; these provide controlled environments within which workflows can be executed. Environments can also be managed in a more lightweight fashion using environment variables and per-process namespaces.
iii. *Managing workflows*. This is commonly handled with *workflow engines* (e.g., Toil [8], SnakeMake [9], WDL [10], Cromwell [11], NextFlow [12], Ruffus [13], and Rubra [14]), which manage *stages*[1] and their execution, providing features like parallelism, remote building, resumability, and logging.

Some tools tackle more than one of these challenges: Conda, for example, began life as a Python package manager, but more recently aims to manage both software and environments in a language-agnostic way [4].

All of these challenges need to be addressed *at scale*: bioinformatics workflows are computationally demanding, and often need to be executed on computing clusters, on remote computing farms, or in the cloud. The combination of technologies used to address these challenges are called a *reproducibility stack* by Grüning, Chilton, Köster, et al. [15].

Our contributions. We present BioNix, a lightweight library that cleanly deals with all three of these challenges within the one system.

Two aspects of BioNix's design enable these improvements.

1 We define a *stage* as the concrete execution of one or more executables on one or more input files, producing one or more output files.

The first is that BioNix is built on *Nix*, a next generation cross-platform software deployment system. The second is that in BioNix, stages of a workflow are modelled as *pure functions* – i.e., functions that are free of side effects: workflow stages cannot modify shared state, and so are extremely modular.

These design choices give BioNix several novel features, which we explain using the complete workflow and associated build graph depicted in Example 1:

i. BioNix *manages both software and workflows within the one system*. The build graph in Example 1 has nodes corresponding not just to workflow stages and inputs, but also to software dependencies.
ii. Each stage of a BioNix workflow *implicitly specifies its entire computational environment*. Dependencies are tracked down to the kernel level, and each stage is executed in its own sandbox, resulting in strong reproducibility guarantees and obviating the need for containers[2]. In Nix, sandboxing is enabled by default and may be explicitly disabled for either any specific build or globally.
iii. Nix tracks the entire tree of runtime and build time dependencies with *fine grained versioning*. In the example pipeline this means that not only the version of `bwa`, but also the specific versions of `gcc` and `bash` under which `bwa` was compiled, are captured. All of these versions are fixed by specifying which versions of BioNix and `Nixpkgs` we use (by their commit hashes): the code on the right forms a fully reproducible specification of the associated workflow.
At the same time, it is straightforward to specify specific versions of software for distinct stages, and to use distinct versions of a given piece of software in parallel.
iv. BioNix uses *a simple, purely functional domain-specific language* – the Nix expression language – for specifying workflows. Constructing a workflow is reduced to function composition; stages, workflows, and software dependencies are all represented as pure functions from dependencies to outputs. Because of purity, stages are guaranteed not to influence each other except through their inputs and outputs, and so can be safely recombined. An example of this in Example 1 is our ability to compose workflow steps using the higher-order `map` and `pipe` functions.
The Nix expression language can be considered a compromise between the safety of static configuration files and the expressiveness of a general purpose programming language. Configuration files are predictable, but writing them can involve a lot of boilerplate and repetition. General purpose programming languages allow one to abstract away much of this verbosity, but at the cost of some safety and predictability, for example when they allow workflows to modify unrelated parts of the filesystem. Domain-specific languages like Nix aim to be sufficiently expressive without the error-prone power of a general purpose programming language.

BioNix includes many features found among the most powerful existing workflow managers. Intermediate files do not need to be named or managed. Multiple versions of the same piece of software can be used simultanously. BioNix workflows are automatically parallelisable, can be executed in High Performance Computing (HPC) environments or in the cloud, and are fully resumable in cases of interrupted execution. BioNix also allows for conditional execution: that is, different stages may be executed depending on previous stages' outputs.

BioNix includes the following components over and above base Nix:

i. A framework for specifying workflows in the Nix expression language.
ii. A library containing some commonly used bioinformatics tools and helpful workflow specification utilities.
iii. A module allowing workflows to be executed on HPC clusters.
iv. Basic typing to capture metadata and prevent invalid workflow specifications.

The rest of the paper first explains the basics of the Nix system and associated expression language. Next, we describe the design and implementation of BioNix. Finally, we describe an example workflow, and compare Bionix with existing bioinformatics workflow managers.

Preliminaries

The Nix deployment system

The Nix deployment system emerged from the work of Dolstra [16] and Dolstra, Jonge, and Visser [17]. Nix was originally designed as a software package manager, but has since been adapted to managing OS configurations (the `NixOS` project [18, 19]). BioNix represents a further extension of Nix to manage bioinformatics workflows.

The Nix system has three main components:

i. *Build products* or *outputs* may be any kind of directory, file, or collection of files. When using Nix as a traditional package manager, the build products typically consist of the compiled binaries and libraries associated with an application. In our case, build products are any output associated with a bioinformatics workflow or stage.
ii. *Derivations* are static configuration files (ending in `.drv`) that specify all of the inputs and procedures required to produce a given build product. If a build product has prerequisites, then its derivation will refer to the derivations corresponding to those prerequisites.
iii. *Nix expressions* are written in a simple, high-level domain-specific language designed for specifying and manipulating derivations. Derivations are represented in the Nix language as collections of name-value pairs – similar to JSON objects – called "sets." Nix expressions may also make use of various built-in design patterns to provide further extensibility and flexibility.

The basic build process in Nix is as follows: a Nix expression is *instantiated* to yield a tree of *derivations* describing how to generate the associated *build products*. Derivations are then *realised* by the build system to produce the build products themselves. Nix expressions, derivations, and build products are somewhat analogous to source code, object files, and compiled binaries.

The Nix expression corresponding to a given build product will generally take the form of a pure function from dependencies to the corresponding output derivation. Using ML-style notation for types, one can represent this as

$$\text{Dependencies} \rightarrow \text{Output}.$$

Nix ensures that derivations are precisely specified by giving both derivations and build products hash-based names. The hash of a derivation is a function of all of the steps required to produce the associated build product, as well as the hashes of all of its dependencies.

The *Nix store*, usually located on the filesystem at `/nix`, provides a single, flat namespace for all derivations and build prod-

2 Containers and static binaries can still be used in a BioNix workflow if required, but they are generally avoided to reduce side effects.

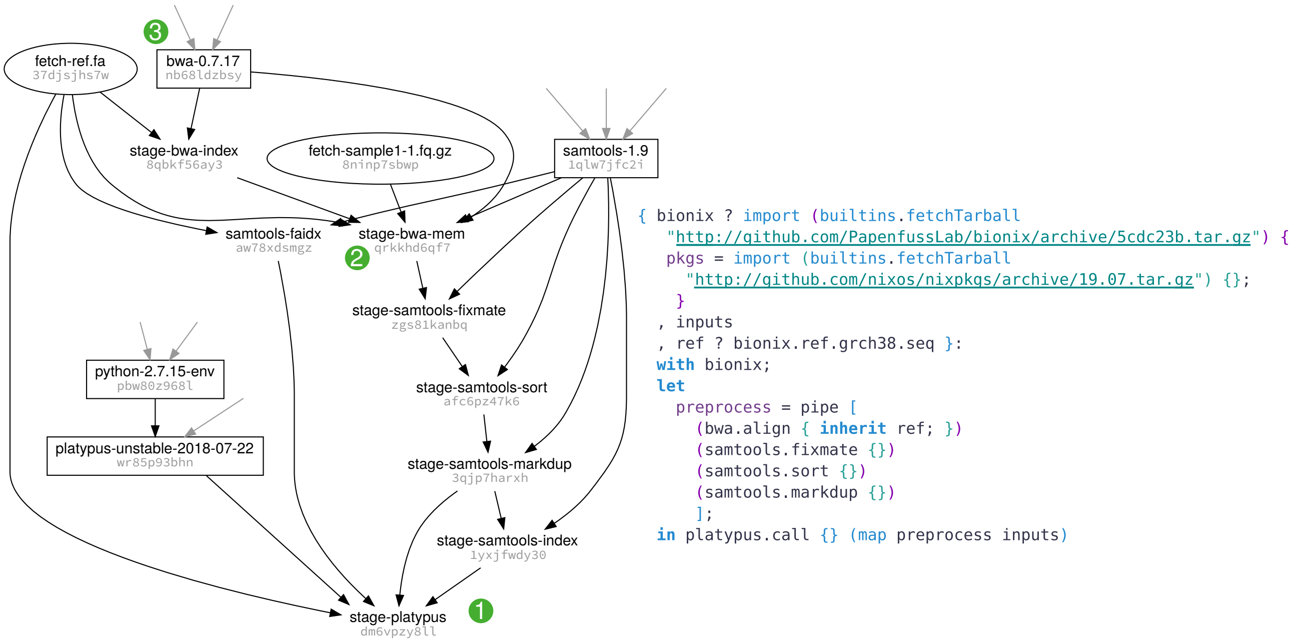


```
{ bionix ? import (builtins.fetchTarball
    "http://github.com/PapenfussLab/bionix/archive/5cdc23b.tar.gz") {
    pkgs = import (builtins.fetchTarball
      "http://github.com/nixos/nixpkgs/archive/19.07.tar.gz") {};
    }
, inputs
, ref ? bionix.ref.grch38.seq }:
with bionix;
let
  preprocess = pipe [
    (bwa.align { inherit ref; })
    (samtools.fixmate {})
    (samtools.sort {})
    (samtools.markdup {})
    ];
in platypus.call {} (map preprocess inputs)
```

Example 1. An example workflow specified in BioNix (right) with a portion of the resulting build graph (left). In the build graph, rectangular nodes correspond to software dependencies and elliptical nodes to data files. Grey arrows indicate dependencies that are not illustrated in the figure. The workflow on the right corresponds to the terminal node in the build graph annotated with (1). The node annotated (2) in the graph corresponds to a single stage in workflow and the corresponding BioNix code can be found in Example 4. The final node annotated (3) corresponds to a software dependency provided by `Nixpkgs` with the corresponding code in Example 6.

ucts and is writable by only the Nix system. Users typically access the store through *environments*: organised collections of soft links exported to `$PATH`.

The Nix community maintains online repository of prebuilt software called `Nixpkgs` [20], which contains over 40,000 software packages.

The Nix expression language

We briefly introduce those parts of the Nix expression language required to understand the rest of the paper.

Sets are the most important datatype in Nix, and correspond to what are sometimes called associative arrays, records, or dictionaries in other languages. Set elements can be accessed by name: `{ a=1; }.a == 1`.

Lists are delimited by square brackets and may contain elements of heterogeneous types separated by whitespace – for example, `[1 2 3 ``a'' ``b'' ``c'' true false]`.

The Nix language makes heavy use of *anonymous functions* (also called *lambda expressions*). The following denotes a function that increments its argument: `x: x + 1`. Nix does not support functions of multiple arguments; instead, it is common for functions to take a set as input. This is written `{ a, b, c, …}: …`. Nix allows defaults to be provided for some elements, which are used if the function is called without providing the element. This is denoted using a question mark: the function `{ a ? 5 }: …` will by default assign `a` the value `5`. Alternatively, one can mimic multi-argument functions using *higher-order* functions – i.e., functions which return functions. For example, `x: y: x + y` denotes a function that adds its two arguments together.

Values can be bound to variable names using the `let … in …` construction. We could bind the example above to a name and then invoke it on some parameters: `let f = x: y: x + y; in f 1 2`. Function application is denoted with whitespace (with lower precedence than list elements) and associates to the left; for example `a b c` denotes `(a(b))(c)`. *Pattern matching* allows simultaneous binding of elements contained in a set: `let {a, b} = {a = 1; b = 2;}; in a + b == 3`. Finally, the `with x; …` construction brings the field names of a set `x` into scope in the subsequent expression.

Implementation

The BioNix library itself is designed as a tree of functions, with each function representing one *stage* of processing. The BioNix tree follows the pattern of `Nixpkgs`; bioinformatics software (e.g., `bwa`, `samtools`, etc) forms the top level, and stages based on subcommands form the second level (e.g., `bwa.align`). As in `Nixpkgs`, defaults can be overridden throughout the whole tree easily.

We will step through three examples of (slightly simplified) BioNix code that generates the build graph in Example 1: the workflow specification, a stage specification, and an expression for a software dependency.

Specifying a workflow

Example 1 shows a simple variant calling workflow using BWA [21, 22] for alignment, samtools [23] for sorting and duplicate marking, and platypus [24] for variant calling. The whole workflow is a single anonymous function, taking dependencies and inputs – the set spanning the first 8 lines – to an output (the final line):

$$\text{(Inputs, Options, \& Dependencies)} \rightarrow \text{Output.}$$

The output of this workflow is the output of platypus, which is a `.vcf` file.

One of the dependencies of the workflow is BioNix itself. If

the user does not specify a version to use, the workflow defaults to using the specific commit indicated. Similarly, if the user does not specify a reference, the workflow defaults to GRCh38. Fixing a version of BioNix and Nixpkgs automatically fixes versions of all software used in the pipline, though these can be individually specifically altered if desired (see Example 2 and Example 3).

```
let
  oldnix = import (fetchFromGitHub {
    owner = "NixOS";
    repo = "nixpkgs";
    rev = "83a893c38a83877588e3ca7ccfeabaa973c30acd";
    sha256 = "0q7214hag7h95irvhkdb648m09b9jspb0raw1qjrx7y4grzb165h";

  }) { };

  jre = oldnix.openjdk7;

in ...
```

Example 2. This example is an extract from the MuTect stage and demonstrates how specific software versions can be referenced. Here the deprecated JDK 7 required by MuTect is accessed through an old revision of Nixpkgs.

Each stage – for example, `bwa.align`, `samtools.fixmate`, or `platypus.call` – is represented by a higher-order function that takes options and dependencies, and returns a function from inputs to outputs. The type of a stage[3] can be represented as

$$(\text{Options} \,\&\, \text{Dependencies}) \to (\text{Inputs} \to \text{Output}).$$

```
let
  octopus-git = octopus-caller.overrideAttrs (attrs: {
    src = fetchFromGitHub {
      owner = "luntergroup";
      repo = "octopus";
      rev = "f88d35b9b93d11a086eb87bb3722874a3ea5250e";
      sha256 = "171cmpx6x8p1q4d0k9lj2mwcyhhrc20cskqhwdyl6fa1clx40b7r";
    };
  });
in ...
```

Example 3. Nixpkgs has a flexible overrides system that allows derivations to be selectively modified. Here the Octopus variant caller switched to the latest development branch (as of 2020-03-20) instead of the current release.

BioNix dependencies by default include the BioNix tree itself – to allow use of other (sub-)stages – and well as the `Nixpkgs` collection, which provides the necessary general-purose software. For most of our stages we do not pass in any additional options, and so the first argument is `{}`. However, `bwa.align` requires that we specify a reference, and so we explicitly pass in the `ref` declared at the beginning of the workflow.

We make use of several helpful abstractions from functional programming. For example, we define a new function, `preprocess`, that takes a sample and performs alignment, mate-fixing, sorting, and duplicate-marking. We also use the `pipe` function in BioNix to sequentially compose a list of functions. Finally, we `map` this function over all our inputs. The Nix expression language allows for this abstraction and modularity without introducing side-effects.

3 Functional programmers will recognise this as a *curried* version of the type of a workflow.

Specifying a stage

```
{ bionix
, ref
, bamOutput ? true
}:

{ input1
, input2 ? null
}:

with bionix;
with lib;

stage {
  name = "bwa-mem";
  buildInputs = with pkgs; [ bwa ] ++
    optional bamOutput samtools;
  buildCommand = ''
    ln -s ${ref} ref.fa
    for f in ${bionix.bwa.index {} ref}/* ; do
      ln -s $f
    done

    bwa mem -t $NIX_BUILD_CORES \
      ref.fa \
      ${fq input1} \
      ${optionalString (input2 != null) input2} \
      ${optionalString bamOutput "| samtools view -b"} \
      > $out
  '';
}
```

Example 4. Specifying an alignment stage using BWA-mem. The expression defines a function mapping parameters (e.g., a choice of reference genome) and the fastq inputs to a derivation produced by the `stage` function. The `stage` function takes as arguments a build script and the requisite software.

Example 4 illustrates an example *stage* in BioNix. In line with our design pattern, the whole stage is represented by an anonymous higher-order function: it takes a record of options and dependencies, and returns a function that takes inputs – in this case, a pair of FASTQ files representing read pairs – and returns a derivation. Notice that we give the reference as part of the first argument to the stage (options and dependencies) rather than as part of the second argument (inputs). This is because often an entire workflow will be parametrised by a single reference genome.

Links are created for both the reference and its associated BWA indices to deal with the standard bioinformatics convention that indices are located in the same directory as the associated indexed file.

Finally, the output is optionally converted to the `.bam` format within the shell script associated with the derivation. BioNix cannot stream data between stages of a workflow: both inputs and outputs of a stage must be a file or set of files.

Example 4 has multiple inputs and only a single output (the BAM file), however multiple outputs are also supported by Nix derivations. Example 5 demonstrates multiple outputs for picard tools [25] where metrics in addition to the main output. The extra output can be accessed via the metrics attribute in the returned derivation.

Specifying a software dependency

For completeness, we also show how to specify a software dependency. In our example workflow, the BWA software is pro-

```
stage {
    name = "picard-markDuplicates";
    buildInputs = with pkgs;
        [ picard-tools ];
    outputs = [ "out" "metrics" ];
    buildCommand = ''
        picard MarkDuplicates \
            I=${inputBam} \
            O=$out \
            M=$metrics
        '';
}
```

Example 5. Extract from the definition of the mark duplicates expression for picard tools demonstrating multiple outputs. The output attribute names the build products, which are assigned unique paths in the store and exposed to the build script via environment variables of the same name.

vided by `Nixpkgs` and Example 6 shows a simplified version of its specification there.

The expression is an anonymous function from dependencies – in this case, the utility libraries `stdenv` and `fetchurl` and the C library dependency `zlib` – to outputs – in this case, the compiled binary for `bwa`. The function body is just a single call to the helper function `mkDerivation`. Since `bwa` follows the first two parts of the common `./configure; make; make install` pattern for building unix software, only the final install phase needs to be specified. Here, the resulting binary is copied into the `bin/` directory.

```
{ stdenv, fetchurl, zlib } :

stdenv.mkDerivation rec {
  name = "bwa-${version}";
  version = "0.7.17";

  src = fetchurl {
    url = "mirror://sourceforge/bio-bwa/${name}.tar.bz2";
    sha256 = "1zfhv2zg9v1icdlq4p9ssc8k01mca5d1bd87w71py2swfi74s6yy";
  };

  buildInputs = [ zlib ];

  installPhase = ''
    install -vD bwa $out/bin/bwa
  '';
}
```

Example 6. Specifying a software dependency for bwa-mem. This is a simplified version of the expression found in `Nixpkgs`. The expression defines the build requirements (zlib) and the steps required to build the software. A standard build process (`configure`, `make`, `make install`) is assumed, so only non-standard commands need to be specified. BWA does not support the standard `make install` for installation, so an install script is defined in the expression.

HPC queue integration

While the Nix build system provides support for both local and remote building, bioinformatics workflows are commonly executed on traditional HPC infrastructure managed by a job schedulers. These systems require users to submit jobs to a queue, along with specified resource limits.

BioNix provides support for queuing systems via a function that takes resource limits and a derivation, and returns a new derivation that will submit the build process as a job to the queuing system instead of building it directly. This design allows arbitrary derivations to be lifted to the queue, and also allows users to combine submission to the queue and building via the Nix build system directly. However, as submission is a (relatively benign) side effect, builds cannot be realised using sandboxing. This is because the default sandbox prevents the build from using software not specified in the expression, and submitting jobs to the scheduler requires interacting with the daemon running outside the build environment. This restriction only applies to cluster execution; local and remote builds fully support sandboxing.

Failures in the queue are handled similarly to execution failures: the build is aborted and reported to the user. This includes when jobs are terminated due to resource limits. The jobid of the submission is recorded in the build log along with any output produced by the job to aid the user in tracing the error.

Tracking types of build products

BioNix gives build products optional *types* in order to prevent errors in workflow specification and to track useful metadata such as the reference used for an alignment. This is a lightweight version of the approach taken by *Bioshake* [26, 27]. Types are implemented as an abstract data type (ADT) and are tracked using Nix's passthru features.

Discussion

Real world use of BioNix

Small variant calling workflow. We have used BioNix to manage a workflow that performs somatic variant calling and Copy Number Variant (CNV) calling on whole genome deep sequencing human data using Minimap2 [28] for alignment, samtools [23] for sorting and marking duplicates, Strelka [29] for somatic variant calling, and CNVkit [30] for CNV calling.

This workflow was executed on HPC infrastructure managed with the TORQUE resource manager [31] using the extensions presented earlier. A total of 1.1TB of (compressed) fastq input was processed, producing 755GB of results (including alignments). The workflow is detailed in Example 7 and a full example executing the workflow on a publicly available melanoma dataset [32] is available in the BioNix repository.

Structural variant calling at scale. We have also used BioNix to execute a workflow that processes 6.8TB of whole genome sequencing data from mice, performing quality checking, alignment, and merging, and structural variant calling using `gridss` [33] with a range of parameters. This resulted in a total of 5.3TB of results. See Example 8 for the workflow used.

Limitations of BioNix

BioNix leverages the underlying Nix system to achieve its reproducibility, and consequently is subject to the same limitations present in Nix.

A given stage may only write to the store location assigned to it, so streaming data between two distinct stages is not possible. Streaming steps must therefore be combined into one stage, which can be constructed with higher order functions. Streaming between two independent builds would be difficult to support: the distributed design of Nix implies that different builds may be executing on independent machines.

Nix and BioNix will currently rebuild an output unnecessarily when dependencies of its inputs have changed, but the

```
{bionix ? import <bionix> {}, pair, fetch}:

with bionix;
with lib;
with types;

with minimap2;
with samtools;
with snpeff;

let
  preprocess = s: pipe s [
    fetch
    (align { preset = "sr"; ref = ref.grch38.seq; flags = "-R'@RG\\tID:${s.type}\\tSM:${s.type}'"; })
    (fixmate {})
    (sort { })
    (markdup { })
  ];

  dropErrors = input: stage {
    name = "drop-errors";
    buildCommand = ''
      grep -v "ERROR_" ${input} > $out
    '';
    passthru.filetype = input.filetype;
  };

  bams = mapAttrs (_: preprocess) pair;

  variants = let
    somatic = strelka.callSomatic { } bams; in mapAttrs (_: flip pipe [
      (compression.uncompress { })
      (snpeff.annotate { db = ref.grch38.snpeff.db; })
      dropErrors
      (snpeff.dbnsfp { dbnsfp = ref.grch38.snpeff.dbnsfp; })
    ]) {
      "snvs.vcf" = somatic.snvs;
      "indels.vcf" = somatic.snvs;
      "germline.vcf" = strelka.call { } [bams.normal];
    };

  cnvs = cnvkit.callCNV { } { normals = [ bams.normal ]; tumours = [ bams.tumour ]; };

in linkOutputs {
  inherit variants;
  alignments = linkOutputs (mapAttrs' (n: nameValuePair (n + ".bam")) bams);
  cnvkit = cnvs;
}
```

Example 7. The tumour-normal small variant calling workflow used for calling variants on clinical samples. Reads are aligned using Minimap2 [28], variants called using Strelka [29], and finally CNVs with CNVkit [30]. The inputs are a pair of samples (as an attribute set containing `normal` and `tumour` attributes), a method `fetch` for fetching the reads associated with a given sample, and BioNix.

```
{ bionix, baseUrl, mice ? import ../metadata/mice.nix }:

with bionix;
with lib;

let
  # Utility function
  update = f: x: x // (f x);

  # Process fastqs ############################################
  fetch = { filename, sha256sum, ... }:
    fetchFastQGZ {
      url = baseUrl + filename;
      sha256 = sha256sum;
    };

  updateFastq = update (fq: { fastqcOutput = fastqc.check { } (fetch fq); });

  # Process sample run ########################################
  fetchInputs = { fq1, fq2, ... }: {
    input1 = fetch fq1;
    input2 = fetch fq2;
  };

  alignSortSampleRun = sr:
    pipe sr [
      fetchInputs
      (bwa.align {
        ref = ref.grcm38.seq;
        flags = "-R'@RG\\tID:${sr.id_col}\\tSM:${sr.sample_id}'";
      })
      (samtools.sort { })
    ];

  updateSampleRun = update (sr: {
    sampleRunBam = alignSortSampleRun sr;
    # recurse
    fq1 = updateFastq sr.fq1;
    fq2 = updateFastq sr.fq2;
  });

  # Process samples ###########################################
  mergedBam = flip pipe [ (map (sr: sr.sampleRunBam)) (samtools.merge { }) ];

  updateSample = update (sample:
    let updated_sample_runs = map updateSampleRun sample.sample_runs;
    in {
      sample_runs = updated_sample_runs;
      mergedBam = mergedBam updated_sample_runs;
    });

  # Process mice ##############################################
  updateMouse = update (mouse: rec {
    gridssCalls =
      gridss.callAndAssemble (map (sample: sample.mergedBam) samples);
    samples = map updateSample mouse.samples;
  });

in map updateMouse mice
```

Example 8. Structural variant calling for a mouse dataset. Stages include quality checking with FastQC [34], alignment with BWA [21], merging with samtools [23] and finally structural variant calling with GRIDSS [33]. The inputs to the expression are BioNix, a base URL where the fastq files can be found, and the metadata describing the experimental design, sequencing data and hashes. This workflow is structured so that metadata are “annotated” with build products, analogous to building up a data structure in a general-purpose language. Since Nix is lazy, the build products will only be built when requested. Comparing with 7 shows how flexibly workflows can be specified within BioNix.

inputs themselves have not. The reason is that the Nix store is not content addressed; store locations are based on the cryptographic hash of all inputs used in building an output, rather than the output itself. This has been referred to as an *extensional* model [17]. The proposed *intensional* store model [17] introduces content addressable storage and hash rewriting, allowing better sharing of components and reducing unnecessary builds. This feature is currently under implementation and is not available in the latest Nix release (2.3.1).

The Nix language, though extremely simple, has an idiosyncratic syntax that draws from both curly brace and functional programming languages; some may find this unfamiliar or offputting.

Finally, Nix does not have an advanced type system. BioNix provides type safety for many of its stages through an implementation of ADTs, but as these data types are implemented in Nix itself the error reporting can be obscure.

Related work

We discuss here two categories of work related to our own. The first consists of other projects making use of the Nix deployment system to manage data processing workflows; the second concerns existing workflow management tools popular in bioinformatics and computational biology.

Similar adaptations of the Nix system

Several groups have made use of Nix to manage the *environments* in which computational workflows are executed. Researchers at GRICAD at the Université Grenoble Alpes have made use of Nix as an HPC package management system [35, 36]. The Pipelines in Genomics (PiGx) project [37] uses Guix – an implementation of the Nix system using GNU Scheme in place of the Nix expression language – to produce a set of reproducible "turn-key" workflows for bioinformatics and computational biology, configured via simple static config files. Similar uses of Nix for reproducible research have also been suggested by Blair Archibald of the Software Sustainability Institute [38, 39] and Bruno Vieira at the Mozilla Foundation [40].

However, none of these approaches use Nix to specify workflows themselves; instead, Nix is used as a replacement for package managers and containers. BioNix takes the next step and embeds the workflows into the Nix system.

Two projects that we know of make use of Nix to manage workflows themselves: Mix, a Nix-based system for specifying data processing pipelines developed at SoundCloud [41], and Fractalide, a service programming platform using dataflow graphs [42].

Mix is built on the `hnix` project [43] and implements a new builder dedicated to data workflows. Mix redefines derivations to remove the Nix store and allow storage of products on a distributed file system. Consequently, Mix cannot take advantage of `Nixpkgs` and focuses entirely on the workflows, without capturing the associated computational environments.

Fractalide is an effort to provide a dataflow graph programming platform with an initial focus on microservices and the internet of things. Though it builds on Nix, it also extends the base language with a new language for specifying the dataflow graphs, and relies on bindings to other languages to provide an interface to the actual data processing (i.e., the microservice). By contrast, BioNix focuses on Bioinformatics workflows, is implemented entirely within the existing Nix ecosystem, and calls existing pieces of software via their command line interfaces.

The Guix Workflow Language (GWL) [44, 45] is in many ways the workflow manager closest in approach to BioNix. GWL, like PiGx, is built on Guix, and so inherits the reproducibility guarantees of a Nix-like system. Unlike PiGx, GWL manages workflows themselves using Guix, rather than using it only to provide the necessary software environment. However unlike BioNix, stages in GWL are not represented by functions but by data structures; workflows are specified via manual construction of the associated build graph; and workflow stages are untyped.

Existing workflow managers for computational biology

There are a large number of existing workflow systems in use today [46, 47]. A complete review of the existing systems is out of scope of this paper, however we will briefly review the more commonly used systems [48].

Leipzig [48] categorises workflow systems into two main categories: implicit or explicit syntax. Those with explicit syntax detail the workflow between stages explicitely; BioNix would fall into this category as our modelling of a workflow as function composition explicitly links the steps together into a workflow, as does Workflow Description Language (WDL) [10]. The other category, implicit syntax, are those systems whereby the stages are connected through abstract rules linking stages with their dependencies. Examples of implicit workflows are SnakeMake [9] and Nextflow [12]. We choose a representative selection of these broad categories in the en suite.

As already mentioned, current best practice aims at reproducibility using a combination of package managers, containers, and workflow engines. BioNix combines the functionality of all of these, and in this sense is difficult to compare with existing workflow management tools.

However, we can compare the syntax of BioNix with that of existing workflow managers by implementing toy piplines in each. Examples 9 and 10 illustrate two simple examples from the documentation of WDL [10] and NextFlow [12] alongside the equivalent BioNix expression. BioNix necessarily defines the software used in the execution of the workflow, and software outside of Nix is unavailable. By contrast, the WDL and NextFlow examples presented are valid workflow specifications without software definitions, though they do support software environment management via containers (e.g., docker) or package managers (e.g., conda). Example 11 demonstrates support for docker in WDL.

BioNix might also be compared with Common Workflow Language (CWL) [52], which is a standard specification language intended for describing workflows in a portable way. However, CWL is increasingly used as a target for other build systems, rather than being written directly. In this sense, CWL increasingly plays a role similar to Nix's derivation files, which are complete, portable, machine-readable specifications that can be built on local or remote systems.

Galaxy [53] is a popular workflow platform that provides a web-based GUI for specification of workflows and execution controls. Galaxy provides facilities to manage the computational environment via various package management tools, with Conda being popular. Nix can be integrated into Galaxy, which would allow Galaxy to leverage the strong reproducibility guarantees of Nix [16, 17]. This would be similar to the approach taken by PiGx.

Cuneiform [54, 55] is a functional programming language for large-scale data analysis workflows. In Cuneiform, as in BioNix, workflow stages are modelled as pure functions. Cuneiform also has an elegant foreign function interface (FFI), allowing the seamless use of code snippets from a variety of languages – bash, Python, R, and others – as well as a language-level static type system. On the other hand, Cuneiform does not manage software dependencies, and so lacks the reproducibility guarantees that BioNix leverages from Nix.

Finally, SciPipe [56] is a recent workflow library that focuses on dynamic execution and streaming. Like Bionix, SciP-

```
{ bionix ? import <bionix> {} }:

with bionix;
with lib;

let

  prepare = splitString "\n" (removeSuffix "\n" (readFile (stage {
    name = "prepare";
    buildInputs = [ pkgs.python3 ];
    buildCommand = ''
      python -c "print('one\ntwo\nthree\nfour', end='''')" > $out
    '';
  })));

  analysis = str: removeSuffix "\n" (readFile (stage {
    name = "analysis";
    buildInputs = [ pkgs.python ];
    buildCommand = ''
      python -c "print('_${str}_')" > $out
    '';
  }));

  gather = strs: stage {
    name = "gather";
    buildCommand = ''
      echo ${concatStringsSep " " strs} > $out
    '';
  };

in gather (map analysis prepare)
```

```
task prepare {
  command <<<
    python -c "print('one\ntwo\nthree\nfour')"
  >>>
  output {
    Array[String] array = read_lines(stdout())
  }
}

task analysis {
  String str
  command <<<
    python -c "print('_${str}_')"
  >>>
  output {
    String out = read_string(stdout())
  }
}

task gather {
  Array[String] array
  command <<<
    echo ${sep=' ' array}
  >>>
  output {
    String str = read_string(stdout())
  }
}

workflow example {
  call prepare
  scatter (x in prepare.array) {
    call analysis {input: str=x}
  }
  call gather {input: array=analysis.out}
}
```

Example 9. Verbatim scatter-gather example from WDL [49] documentation with the BioNix implementation on the left and WDL on the right. The workflow generates some input data using python, parses it into lines, transforms each line via a simple python script, then collects all lines into a final output. It is unusual to parse and split using the Nix language – typically this would instead be done through a build – but we have done so to maintain a closer translation of the WDL example. In BioNix we must specify the python dependency: as the entire software environment is managed, a failure to specify software will result in a failed build. The BioNix example also shows how different software versions can be combined: Python 3 is used in the `prepare` stage, but Python 2 is used in the `analysis` stage.

```
{ bionix ? import <bionix> {}
, input ? ./sample.fa}:

with bionix;
with lib;

let
  splitSequences = fa: stage {
    name = "splitSequences";
    buildInputs = [ pkgs.gawk ];
    buildCommand = ''
      awk '/^>/{f="seq_"++d} {print > f}' ${fa}
      mkdir $out
      cp seq* $out
    '';
  };

  reverse = fa: stage {
    name = "reverse";
    buildCommand = ''
      ${pkgs.utillinux}/bin/rev ${fa} > $out
    '';
  };

in pipe [
  splitSequences
  (each reverse)
] input
```

```
params.in = "$baseDir/data/sample.fa"
sequences = file(params.in)

/*
 * split a fasta file in multiple files
 */
process splitSequences {

    input:
    file 'input.fa' from sequences

    output:
    file 'seq_*' into records

    """
    awk '/^>/{f="seq_"++d} {print > f}' < input.fa
    """

}

/*
 * Simple reverse the sequences
 */
process reverse {

    input:
    file x from records

    output:
    stdout result

    """
    cat $x | rev
    """
}

/*
 * print the channel content
 */
result.subscribe { println it }
```

Example 10. Nextflow basic pipeline example [50] (right) taken verbatim from the documentation and translated to BioNix (left). The example splits a single FastA file into a collection of FastA files, each containing exactly one sequence. The sequences are then reversed (in parallel) and then gathered back into one file in the final step. The BioNix `pipe` function implements reverse function composition for the easy specification of sequences of stages. The BioNix expression requires us to specify which `awk` implementation to use; here we chose GNU Awk.

```
task hello_world {
  String name = "World"
  command {
    echo 'Hello, ${name}'
  }
  output {
    File out = stdout()
  }
  runtime {
    docker: 'ubuntu:latest'
  }
}

workflow hello {
  call hello_world
}
```

```
{ bionix }:

bionix.stage {
  name = "hello";
  str = "World";
  buildCommand = ''
    echo "Hello, $str" > $out
  '';
}
```

Example 11. WDL example using docker for software management (left) taken verbatim from the Cromwell documentation [51] and the equivalent BioNix expression describing the build (right). The software in WDL is fixed by specifying which docker container to use (which could be referred to with a specific hash), while in the BioNix example the software is fixed when a concrete `bionix` is passed to the function.

ipe provides logs at the resolution of each build and allows incremental (partial) builds. On the other hand, SciPipe has a strong focus on streaming which is not supported in Nix between independent builds (see).

Conclusions

We have presented BioNix, a framework built on Nix in which workflows are specified using pure functions. BioNix captures software versions and dependencies, manages computational environments, and composes the various stages of workflows all within the one framework and language. Previous approaches to computational reproducibility have relied on a combination of technologies such as containers, package managers, and workflow engines to achieve the same ends. BioNix unites these functionalities under the one framework, making it simple to specify computational biology workflows with strong reproducibility guarantees.

BioNix is available at http://github.com/PapenfussLab/bionix under the 3-clause BSD license.

Acknowledgements

Thanks to Ramyar Molania and Jocelyn Sietsma Penington for being early adopters of BioNix, and to Ramyar for helpful comments on the manuscript. Thanks to Alan Rubin for many helpful discussions and comments on the manuscript.

A.T.P. was supported by the Lorenzo and Pamela Galli Charitable Trust and by an Australian National Health and Medical Research Council (NHMRC) Program Grant (1054618) and NHMRC Senior Research Fellowship (1116955). The research benefitted by support from the Victorian State Government Operational Infrastructure Support and Australian Government NHMRC Independent Research Institute Infrastructure Support.

J.B. was supported by the Stafford Fox Medical Research Foundation.

Competing interests

The authors declare that they have no competing interests.

Availability of supporting source code and requirements

Project name: BioNix
Project home page: https://github.com/PapenfussLab/BioNix
Operating system(s): NA
Other requirements: Nix
RRID: RRID:SCR_017662
BioTools: biotools:BioNix

References

1. Justin B, Leon DSS, and Anthony PT. Supporting data for ”Unifying package managers, workflow engines, and containers with BioNix for computational reproducibility”. 2020. DOI: 10.5524/100782.
2. Reality check on reproducibility. Nature 2016;533:437–7.
3. Challenges in irreproducible research. Nature, 2018.
4. Package, dependency and environment management for any language—Python, R, Ruby, Lua, Scala, Java, JavaScript, C/ C++, FORTRAN. 2018. URL: https://conda.io/docs/.
5. Grüning B, Dale R, Sjödin A, et al. Bioconda: sustainable and comprehensive software distribution for the life sciences. Nature Methods 2018;15:475–6.
6. Enterprise container platform. 2018. URL: https://www.docker.com.
7. Singularity. 2018. URL: https://www.sylabs.io/singularity/.
8. Vivian J, Rao AA, Nothaft FA, et al. Toil enables reproducible, open source, big biomedical data analyses. Nature Biotechnology 2017;35:314–6.
9. Koster J and Rahmann S. Snakemake–a scalable bioinformatics workflow engine. Bioinformatics 2012;28:2520–2.
10. WDL | Home. URL: https://software.broadinstitute.org/wdl/ (visited on 01/23/2019).
11. URL: https://github.com/broadinstitute/cromwell (visited on 06/17/2020).
12. Di Tommaso P, Chatzou M, Floden EW, Barja PP, Palumbo E, and Notredame C. Nextflow enables reproducible computational workflows. Nature Biotechnology 2017;35:316–9.

13. Goodstadt L. Ruffus: a lightweight Python library for computational pipelines. Bioinformatics 2010;26:2778–9.
14. URL: https://github.com/bjpop/rubra (visited on 06/17/2020).
15. Grüning B, Chilton J, Köster J, et al. Practical Computational Reproducibility in the Life Sciences. Cell Systems 2018;6:631–5.
16. Dolstra E. The Purely Functional Software Deployment Model. PhD thesis. Faculty of Science, Utrecht, The Netherlands, 2006.
17. Dolstra E, Jonge M de, and Visser E. Nix: A Safe and Policy-Free System for Software Deployment. In: *Proceedings of the 18th Large Installation System Administration Conference* (Atlanta). 2004.
18. Dolstra E, Löh A, and Pierron N. NixOS: A Purely Functional Linux Distribution. Journal of Functional Programming 2010:577–615.
19. NixOS. 2019. URL: https://www.nixos.org/nixos.
20. nixpkgs. 2019. URL: https://www.nixos.org/nixpkgs.
21. Li H. Aligning sequence reads, clone sequences and assembly contigs with BWA-MEM. 2013. arXiv: q-bio/1303.3997.
22. Li H and Durbin R. Fast and accurate short read alignment with Burrows-Wheeler transform. Bioinformatics 2009;25:1754–60.
23. Li H, Handsaker B, Wysoker A, et al. The Sequence Alignment/Map format and SAMtools. Bioinformatics 2009;25:2078–9.
24. Rimmer A, Phan H, Mathieson I, et al. Integrating mapping-, assembly- and haplotype-based approaches for calling variants in clinical sequencing applications. Nature Genetics 2014;46:912–8.
25. Picard toolkit. http://broadinstitute.github.io/picard/. 2019.
26. Bedő J. Bioshake: a Haskell EDSL for bioinformatics pipelines. 2018. bioRxiv: http://doi.org/10.1101/529479.
27. Bioshake. 2019. URL: https://github.com/PapenfussLab/bioshake.
28. Li H. Minimap2: pairwise alignment for nucleotide sequences. Bioinformatics 2018;34. Ed. by Birol I:3094–100.
29. Kim S, Scheffler K, Halpern AL, et al. Strelka2: fast and accurate calling of germline and somatic variants. Nature Methods 2018;15:591–4.
30. Talevich E, Shain AH, Botton T, and Bastian BC. CNVkit: Genome-Wide Copy Number Detection and Visualization from Targeted DNA Sequencing. PLOS Computational Biology 2016;12:e1004873.
31. TORQUE Resource Manager. 2019. URL: http://www.adaptivecomputing.com/products/torque/.
32. Cameron DL, Baber J, Shale C, et al. GRIDSS, PURPLE, LINX: Unscrambling the tumor genome via integrated analysis of structural variation and copy number. 2019.
33. Cameron DL, Schröder J, Penington JS, et al. GRIDSS: sensitive and specific genomic rearrangement detection using positional de Bruijn graph assembly. Genome Research 2017;27:2050–60.
34. Andrews S, Krueger F, Segonds-Pichon A, Biggins L, Krueger C, and Wingett S. FastQC. Babraham Institute. Babraham, UK, 2010.
35. Bzeznik B, Henriot O, Reis V, Richard O, and Tavard L. Nix as HPC package management system. In: *Proceedings of the Fourth International Workshop on HPC User Support Tools - HUST'17*. the Fourth International Workshop. Denver, CO, USA: ACM Press, 2017:1–6. DOI: 10.1145/3152493.3152556.
36. Bouttier PA. Nix as HPC package management system. NixCon. 2018.
37. Wurmus R, Uyar B, Osberg B, et al. PiGx: reproducible genomics analysis pipelines with GNU Guix. GigaScience 2018;7.
38. Archibald B. Reproducible Environments With Nix. Software Sustainability Institute. 2017. URL: https://www.software.ac.uk/blog/2017-10-05-reproducible-environments-nix (visited on 01/23/2019).
39. Crouch S, Hong NC, Hettrick S, et al. The Software Sustainability Institute: Changing Research Software Attitudes and Practices. Computing in Science Engineering 2013;15:74–80.
40. Vieira B. A truly reproducible scientific paper? Bruno Vieira. 2017. URL: https://medium.com/@bmpvieira/a-truly-reproducible-scientific-paper-5059b282ee9a (visited on 01/23/2019).
41. Georges Dubus. Mix: Nix for data pipeline configuration. NixCon. London, 2018.
42. Reusable Reproducible Composable Software. 2019. URL: https://github.com/fractalide/fractalide.
43. A Haskell re-implementation of the Nix expression language. 2019. URL: https://github.com/haskell-nix/hnix.
44. Janssen, Roel. Workflow management with GNU Guix. FOSDEM 2017. 2017.
45. Wurmus R. GWL: GNU Workflow Language. FOSDEM 2019. 2019.
46. Pope B. Computational Data Analysis Workflow Systems. 2020. URL: https://github.com/common-workflow-language/common-workflow-language/wiki/Existing-Workflow-systems (visited on 06/02/2020).
47. A curated list of awesome pipeline toolkits inspired by Awesome Sysadmin. URL: https://github.com/pditommaso/awesome-pipeline (visited on 06/02/2020).
48. Leipzig J. A review of bioinformatic pipeline frameworks. Briefings in Bioinformatics 2016:bbw020.
49. Workflow Description Language - Specification and Implementations. 2019. URL: https://github.com/openwdl/wdl/blob/721e16f28f0bf5b3ae8b44df2859b504e10ae13f/README.md#scattergather.
50. Nextflow - Basic pipeline. 2019. URL: https://www.nextflow.io/example1.html.
51. URL: https://cromwell.readthedocs.io/en/stable/tutorials/Containers/#specifying-containers-in-your-workflow (visited on 06/18/2020).
52. Amstutz P, Crusoe MR, Tijanić N, et al. Common Workflow Language, v1.0. 2016. DOI: 10.6084/m9.figshare.3115156.v2.
53. Afgan E, Baker D, Batut B, et al. The Galaxy platform for accessible, reproducible and collaborative biomedical analyses: 2018 update. Nucleic Acids Research 2018;46:W537–W544.
54. Brandt J, Bux M, and Leser U. Cuneiform: A Functional Language for Large Scale Scientific Data Analysis. In: *Proceedings of the Workshops of the EDBT/ICDT*. Vol. 1330. Brussels, Belgium, 2015:17–26.

55. Brandt J, Reisig W, and Leser U. Computation semantics of the functional scientific workflow language Cuneiform*. Journal of Functional Programming 2017;27.
56. Lampa S, Dahlö M, Alvarsson J, and Spjuth O. SciPipe: A workflow library for agile development of complex and dynamic bioinformatics pipelines. GigaScience 2019;8.

Click here to access/download
Supplementary Material
bwa-drv.pdf

Click here to access/download
Supplementary Material
bwa-stage.pdf

Click here to access/download
Supplementary Material
gigascience-logo.pdf

Click here to access/download
Supplementary Material
graph.pdf

Click here to access/download
Supplementary Material
jre.pdf

Click here to access/download
Supplementary Material
mouse.pdf

Click here to access/download
Supplementary Material
nextflow-nix.pdf

Click here to access/download
Supplementary Material
nextflow.pdf

Click here to access/download
Supplementary Material
nix-container.pdf

Click here to access/download
Supplementary Material
oup-contemporary.cls

Click here to access/download
Supplementary Material
override.pdf

Click here to access/download
Supplementary Material
paper.tex

Click here to access/download
Supplementary Material
picard-markDups.pdf

Click here to access/download
Supplementary Material
pipeline.pdf

Click here to access/download
Supplementary Material
references.bib

Click here to access/download
Supplementary Material
stafford.pdf

Click here to access/download
Supplementary Material
wdl-container.pdf

Click here to access/download
Supplementary Material
wdl-nix.pdf

Click here to access/download
Supplementary Material
wdl.pdf

Click here to access/download
Supplementary Material
bwa-drv.svg

Click here to access/download
Supplementary Material
bwa-stage.svg

Click here to access/download
Supplementary Material
default.nix

Click here to access/download
Supplementary Material
graph.svg

Click here to access/download
Supplementary Material
jre.svg

Click here to access/download
Supplementary Material
mouse.svg

Click here to access/download
Supplementary Material
nejm.bbx

Click here to access/download
Supplementary Material
nejm.cbx

Click here to access/download
Supplementary Material
nextflow-nix.svg

Click here to access/download
Supplementary Material
nextflow.svg

Click here to access/download
Supplementary Material
nix-container.svg

Click here to access/download
Supplementary Material
override.svg

Click here to access/download

Supplementary Material

picard-markDups.svg

Click here to access/download
Supplementary Material
pipeline.svg

Click here to access/download
Supplementary Material
shell.nix

Click here to access/download
Supplementary Material
stafford.svg

Click here to access/download
Supplementary Material
tex.nix

Click here to access/download

Supplementary Material

wdl-container.svg

Click here to access/download
Supplementary Material
wdl-nix.svg

Click here to access/download
Supplementary Material
wdl.svg
