# Supplementary material for: Unifying package managers, workflow engines, and containers: Computational reproducibility with BioNix
Source: Gigascience. 2020 Nov 18;9(11):giaa121. doi: 10.1093/gigascience/giaa121 (PMC7672450; doi:10.1093/gigascience/giaa121)
Supplement: giaa121_Response_to_Reviewer_Comments_Original_Submission [file giaa121_response_to_reviewer_comments_original_submission.pdf]

Author's Response To Reviewer Comments

Close

We thank the editor and reviewers for very useful feedback. We have prepared a revised version that addresses the issues raised and strengthens the manuscript. We respond to the individual points in-line.

Editor

> I wish to highlight the comment of reviewer 2 who feels that more
> extensive life-science use cases should be included (in a reproducible
> way, including the data). Open, real-life use cases that demonstrate
> the tool in action are indeed an important feature of our "Technical
> Note" section.

We have added examples to the paper of actual workflow descriptions used, and provided a full real-world example in the repository that processes whole-genome data in a somatic tumour-normal analysis.

> Reviewer 3 feels the presentation of advantages and disadvantages of
> your and similar tools was biased in the present version of the paper.
> I do note that reviewer 3, Johannes Köster, is a co-author of
> "Snakemake", a competitor tool. However, generally speaking, I agree
> with the reviewer's advice that statements on a tool's features and
> advantages/disadvantages should be as factual and unbiased as
> possible.

We apologise for the unintentional bias present and have rewritten the manuscript to add greater discussions around the limitations of our approach.

> Please also register your new software application in the bio.tools
> and SciCrunch.org databases to receive RRID (Research Resource
> Identification Initiative ID) and biotoolsID identifiers, and include
> these in the software availability section your manuscript. This will
> facilitate tracking, reproducibility and re-use of your tool.

We have registered BioNix in the bio.tools registry (biotools:BioNix) and SciCrunch database (SCR_017662). These are now cited in the manuscript.

> Please also ensure that your revised manuscript conforms to the
> journal style, which can be found in the Instructions for Authors on
> the journal homepage.

We have endeavoured to follow the journal style, and would be delighted to address any remaining style issues.

Reviewer #1:

> Novel approach to composition of bioinformatics workflows, targeted to
> developers. Some minor additions to the paper are required: 1). HPC
> environment is mentioned, and the integration with queuing systems,
> how are jobs in the workflow that are dropped by the queuing system
> are handled in bionix ?

We have added a discussion on queue failures to the HPC section, clarifying that queue errors are handled similarly to execution errors: the build is aborted.

> 2). A little more comparison with existing workflow systems is needed
> in the discussion section (there is only a brief mention of Galaxy,
> for the bionix integration with it). How does for example bionix
> compare with nextflow, snakemake etc ? A comparison with points on
> features (present / absent in bionix) to the other workflow platforms
> would be useful in the discussion, for readers looking to compare and
> decide which platform to adopt.

We have expanded the "comparisons" section and included a discussion of Nextflow, along with an explicit syntax comparison. We have also added a "limitations" section that explains the trade-offs involved in BioNix's approach.

We have shied away from a "feature matrix" comparing existing tools for three reasons.

First, our primary contribution—a system that unifies package managers, containers, and workflow tools, using the one abstraction—is less a new feature than novel approach to workflow specification, and we wanted to keep our paper focused on this. (Guix is the closest precedent.)

The second reason is that our approach makes some feature comparisons quite difficult. For example, it is possible to integrate Docker containers into BioNix workflows, and to export workflows in containerised form. But doing so sacrifices the modularity and isolation gained by working within the Nix ecosystem. As such, "Docker support" is not, for us, unambiguously a "feature".

Finally, BioNix itself is less a workflow management system than a thin layer adapting the Nix system to the needs of bioinformaticians. It inherits most of its features from Nix itself—a mature, independent project that is not bioinformatics specific.

> 3). Does bionix integrate with Docker and if not, any disadvantages
> from that ? I believe it should be considered as standard that modern
> bioinformatics workflow systems can run tools prepackaged in
> containers at any of the analysis steps included in the workflow (the
> use of containers is becoming pervasive in bioinformatics for multiple
> reasons). Look for example the nextflow platform, and also Galaxy
> which now enables tool execution from containers.

It is possible to integrate containers into a workflow, as nixpkgs has support for working with Docker and Singularity containers, conda packages, and even binaries. However, using these creates side effects: the construction of the container image is not captured in the Nix specification. Such side effects should be kept to a bare minimum in this system, so container use is discouraged except in the rare cases when no alternative is possible. Our motivating goal is after all to provide a framework that includes computational environment management, obviating the need for containers. We have added a clarifying footnote.

Reviewer #2

> The author describes BioNix, submitted as a technical note. The paper
> is well written, motivates the development well, and discusses
> relevant previous work. Also, we were able to build and run some
> simple examples locally on a Linux laptop. Despite being a technical
> note, my main concern is that we believe that the case studies

> presented in Life Science are very thin.
>
> == Major concerns
>
> Main The authors should present much more in-depth analysis of
> concrete applications in life science. Now the paper is almost
> exclusively a technical description, and the section "Real world use
> of BioNix" is very short and lacks details. No code is presented. If
> more and longer analyses on life science data are available then it
> could be accepted as a Technical Note in Gigascience. Else, I would
> consider this manuscript to be more suited to a more specialized
> technical journal. Further, with such a high focus on reproducibility,
> make sure the readers can reproduce all the cases in the "Real world
> use" section.

We have expanded the real-world section to include the workflow code used to process the examples given. We have not presented any results from the actual analysis of the data as we feel this is outside the scope of the paper.

For the tumour-normal small variant calling workflow, though we cannot release the data we have processed internally using the workflow (it comprises numerous clinical samples and is part of active treatment planning), we have put an example using publicly available melanoma data available from the ENA into the BioNix repository. These data represent a realistic use-case presented in the paper.

The second example workflow, consisting of structural variant calling in mice, has also been presented in the manuscript. However, as the project is unique we haven't been able to find suitable surrogate data to provide a fully executable example. We would be happy to add an additional structural variant calling step to the tumour-normal calling workflow showing how the structural variant calling step scales easily to real-world whole genomes if the reviewers deem it necessary.

> Apart from the main concern, also the following points need to be
> addressed:
>
> 1. There is no section discussing known limitations. All design
> desicions come with certain tradeoffs. Thus for clarity and fairness
> to the reader, we think such a section should be included.

We have added a section to the discussion on known limitations.

> 2. There is one area that could use some more elaboration: The
> handling of multiple inputs and outputs. This is a common case in many
> types of workflows, and in some cases two outputs from one process
> need to take different downstream routes, such as when splitting a
> dataset into a test- and a training set for machine learning.

We have added an example of picard tools producing multiple outputs (the BAM and metrics) to the section. Your example of test and training splits would fit into this with the training data forming the main output and the test data forming an additional output. The test data could then be referred to explicitly in a later stage.

> 3. The examples lack an overview describing what happens in each
> example. This makes it a bit hard to know what to expect from the
> examples.

We have expanded the captions to include an overview of the example in addition to more expository text.

> 4. An area that can often get cumbersome in some cases is where only
> the dependencies between stages or processes are defined, but not how
> each individual output are connected to downstream processes. A
> discussion of how this issue is handled in BioNix would be very
> helpful.
>
> 5. Consider citing the following highly relevant papers:
> - https://www.nature.com/articles/s41592-018-0046-7
> - https://academic.oup.com/gigascience/article/8/5/giz044/5480570

Thank you for the suggestions. We have added bioconda to the discussion of conda. We have also added a discussion comparing our work to scipipe to the manuscript.

> == Minor concerns
>
> Page 1, column 2, 5th row from bottom:
> "the one" perhaps should be "one"?
>
> Page 2, column 1, you say:
> "inflexible verbosity of static conguration files"
> - Is static configuration files really known for verbosity?
> Inflexibility, yes, but verbosity?

We have rewritten this comment to acknowledge the tradeoffs involved in using static configuration files vs. domain-specific languages vs. a general purpose programming language.

> Page 3, column 1, you say:
> "Alternatively, one can mimic multi-argument functions using higher-order functions - i.e., functions which return functions."
> - This is not very clear how that works, and an example or more elaborate explanation would help.

Apologies, that section of the manuscript inadvertantly changed and the example was missing. We have restored the example which should clarify things.

> Page 3, column 2: Broken sentence?
> "For most of our stages we do not pass in any additional options,
> and so the first However"

We have corrected this sentence.

> Page 4, column 2: This sentence needs a little more explanation:
> " However, as submission is a (relatively benign) side effect, builds cannot be realised using sandboxing."

We have clarified the statement.

> Page 5, column 2:
> "written directly"
> Should probably be:
> "written by humans"

We have updated the sentence.

> Page 5, column 2, under "Conclusions"
> "modifying" seems it should be "modify"

We have updated the sentence.

Reviewer #3

> The manuscript describes BioNix, a unified workflow management system
> that uses the nix framework to integrate package management with
> workflow management. The central innovation of BioNix that it finds a
> way to express both the workflow steps as well as software deployment
> steps via the same, functional, Nix DSL.
>
> The paper is written in an understandable and well structured way. The
> central innovation mentioned above is an interesting addition to what
> is available in the field, and indeed deserves to be published.
> Unfortunately, the paper in its current form, in particular when
> comparing with other approaches is sometimes unnecessarily biased and
> not properly justified. It seems like the manuscript tries to convince
> the reader that the presented approach is superior to others. However,
> this claim is never more than a plain hypothesis, as any given
> arguments are not convincing. Instead, I suggest to simply present the
> new approach and maybe briefly compare to others without making a
> subjective judgement. Please find details below.

We have substantially changed the tone of our paper in line with this
suggestion. See below for details.

> # Major comments
>
> The introduction correctly lists three pillars of in silico
> reproducibility. However, there is more to it. First, scalability is
> an issue as well. People need to be able to scale a workflow to their
> computational environment (e.g. cluster or cloud) ideally without the
> need to modify the workflow definition itself. Second, transparency
> and readability is important as well. Apart from computational
> reproduction as a black box, it is important that people can judge
> over the taken steps in terms of parameters and code. Hence, it is
> important that a workflow is in large parts human readable without
> knowing the used definition language.

Thank you for pointing out an interesting aspect. We agree that
scalability is paramount, and that the definition of a workflow needs to
be both readable and independent of how the execution takes place. We
have added a discussion of this to the introduction.

That said, we feel that scalability is a consequence of reproducibility
(and compositionality) rather than a component of it: when the stages of
a workflow are easily reproduced anywhere—e.g., remotely, in the cloud,
or as part of a computing queue—the workflow is inherently scalable.

On readability: we believe that modelling workflows with simple function
composition substantially increases readability. That said, claims about
readability can be quite subjective, and dependent on the programming
languages and paradigms that users are already familiar with. We believe
that our side-by-side syntax comparisons, and our "limitations" section,
now adequately address this.

> Page 2: sandboxing is indeed a nice feature, which, as the authors
> correctly conclude, can also be achieved with containers. The authors
> should clarify whether it has to be enabled/disabled globally or if it
> is possible to change this per step. Also, they should highlight more
> prominently that BioNix only supports it for local (or cloud?)
> execution, not for cluster execution.

We have clarified that the sandboxing can be disabled either globally or
per build. We have also clarified the no-sandbox restriction only

applies to cluster execution and that remote building fully supports
sandboxes.

> Page 2: it is first mentioned that the nixpkgs import fixes the
> versions of all used software, and later the authors mention that
> software versions can be defined per stage. The authors should provide
> an example of the latter.

We have added examples 2 and 3 to the manuscript demonstrating a couple
of ways that software can be explicitly versioned.

> Page 2, paragraph 3: the statement "inflexible verbosity of
> configuration files ... error-prone power of general purpose language"
> is fairly judgemental, without providing any reasoning. If the authors
> make such a statement, they need to provide particular reasons in
> detail, and also highlight the upsides of both. Also, an aspect not
> considered here is that there are situations where config files are
> more suitable than a functional language and as well where a general
> purpose programming language is more suitable than a functional
> specification language like Nix. One could argue that this is the case
> for workflow management. It combines the need for declaring options,
> parameters, software dependencies (which configuration file languages
> are designed for), with the need for declaring steps (obviously a
> declarative task) and the need for flexibility in certain corner
> cases, e.g. complex aggregations, conditional execution (for which the
> full power of general purpose programming languages might be needed).
> Hence, there is certainly a case for not unifying all the different
> aspects of workflow definition in a single language. I would like to
> see the paper discussing this openly, without making a judgement. An
> example for such a system is Snakemake, which provides a declarative
> syntax for workflow step definition on top of Python, a general
> purpose language, in combination with YAML for configuration files and
> conda software environment definition files for ensuring controlled
> execution environments. Since Snakemake is among the most widely used
> workflow managers it should definitely be discussed along with Galaxy,
> Nextflow, WDL and CWL here.

We have expanded our discussion of the tradeoffs involved in using
configuration files vs. a domain specific language vs. a general purpose
programming language.

> If understood correctly, the submission of a BioNix workflow to a
> cluster system needs the modification of the workflow definition by
> adding submission functions. This would limit reproducibility (and
> should be communicated as such), because the workflow potentially
> needs to be adapted before execution on a new system.

We have made it clear that this modification can take place through a
single, global override. The tumour-normal small variant calling
workflow in the repository now contains an example of this for
submission to torque or slurm.

We believe that including job submissions in the workflow in this way
lets us both 1) be "up front" about potential side effects associated
with choosing a given job submission system; while at the same time
2) allowing queueing systems to be switched easily and globally.

> The comparison with other systems is extremely biased. See details
> below.

> For example, both Snakemake and Nextflow support streaming between
> jobs and conditional execution, which does not seem to be supported by

> BioNix. It is of course infeasible to list the union of all features
> of all workflow management systems, but (a) this should be clearly
> communicated and (b) one should try to find a balance of advantages
> and disadvantages of all systems, or even better just list available
> and missing features and leave the judgement to the reader.

We have now stated explicitly that BioNix does not support streaming
between stages.

BioNix does support conditional execution, and we have made this clear
in the main text.

> The authors mention that the nextflow definition is more implicit than
> BioNix, and this may increase difficulty of interpretation. First,
> from the example, I cannot see this increased implicitness. Second,
> implicitness can also be an advantage, and explicitness also has
> downsides. Please communicate this openly.

We have removed this comment.

> I could not follow the comparison with CWL. First, I do not agree that
> CWL is low-level. I would agree with a statement saying that CWL is
> quite verbose and hard to read. However, at least for somebody not
> familiar with Nix, this is also the case for BioNix. Readability for
> non-authors is a very important aspect, and both systems are not the
> best I have seen so far in this regard.

We have removed the labelling of CWL as low-level, but have clarified in
the text that CWL is increasingly used as a machine-readable target by
other build systems, and this sense is more analgous to Nix's derivation
format than to BioNix per se.

> The statement that Nix integration in Galaxy would lead to a better
> control of the computational environment has to be justified. What
> makes Nix better than Conda or Containers, in terms of control?

Because of its hash-based tracking of dependencies, Nix allows for—and
in fact enforces—a more precise specification of software versions and
computational environments than is common in either package managers or
containers. We have nonetheless toned down the statements in the
manuscript.

> In the comparison with Guix, the manuscript mixes a statement that I
> would consider neutral (functional vs. data structures) with something
> negative (no types). Maybe try to separate the two, such that
> functional vs DS does not inherit a negative connotation.

We have rewritten the section to remove the negative connotation.

> The conclusion, well, draws the conclusion that BioNix workflows are
> easier to specify, share execute, modify and reproduce. This is a very
> general statement that I do not believe given the arguments in the
> manuscript before. Why is the functional language of Nix easier to
> modify than e.g. a Nextflow or WDL-based workflow definition? In
> particular why is it an advantage to integrate everything into one
> language? For example, speaking about modification: if one just wants
> to modify a software version or a configuration parameter, all one has
> to do with a Snakemake workflow is to edit two YAML files (the
> respective Conda software environment, and the config file). This
> seems easier than with BioNix at first sight.

We have rewritten the conclusion to emphasize 1. the simplicity that

comes with using a single platform/language/abstraction to manage
environments, software, and workflows, and 2. the strong reproducibility
guarantees provided by Nix and BioNix.

> Example 5 compares BioNix with Nextflow and WDL. The caption says that
> the Nextflow pipeline does not capture the software used. I am sorry
> to say this, but such a statement almost upsets me. Nextflow is very
> well able to define this. By adding a single line to each step, one
> could either define a container or an isolated conda environment. I
> suggest to carefully read the documentation of tools before writing
> such comparisons.

We did not intend to convey that Nextflow cannot specify container
images nor Conda environments, meerly that the particular example
presented does not. This is notable because it is not possible in
BioNix: a build that does not completely specify all required software
will never execute successfully. The caption has been revised to clarify
this and we apologise for the misleading phrasing.

> # Minor comments

> Various paragraphs on page 3 are somehow messed up (incomplete
> sentences). Please carefully check them.

> Page 4, right column, first paragraph: scheduler, not schedulerS.

We have corrected this typo.

Close
