# Supplementary material for: Unifying package managers, workflow engines, and containers: Computational reproducibility with BioNix
Source: Gigascience. 2020 Nov 18;9(11):giaa121. doi: 10.1093/gigascience/giaa121 (PMC7672450; doi:10.1093/gigascience/giaa121)
Supplement: giaa121_Response_to_Reviewer_Comments_Revision_1 [file giaa121_response_to_reviewer_comments_revision_1.pdf]

Author's Response To Reviewer Comments

Close

> Your revised manuscript "Unifying package managers, workflow engines,
> and containers: computational reproducibility with BioNix"
> (GIGA-D-19-00324R1) has been re-assessed. It is potentially acceptable
> for publication in GigaScience, once you have addressed some follow-up
> comments by reviewer 3 (see below) - in particular, the reviewer feels
> that the comparison section needs to be more complete, with all major
> workflow management systems named and cited.
>
> Reviewer #3:
>
> First, I'd like to thank the authors for carefully considering my
> previous comments. Some issues remain, which I'd like to address below.
>
> # Major comments
>
> * The comparison with other systems is still quite short. Nevertheless
> it is necessary (also in the interest of the authors) to show that
> they know the field before making claims and developing something new.
> Of course I completely understand the reasoning behind not making
> feature matrices and full comparisons, this is the job of review
> papers. Still, it is good scientific practice to briefly summarize the
> _entire_ field when publishing something new. At least, all major
> workflow management systems should be named and cited (check out this
> for a list: https://github.com/pditommaso/awesome-pipeline). The
> authors could then say that you chose some representatives for
> comparison... The chosen set the authors have now is already quite
> good, because it represents the different niches.
> * Page 2, left part, second-last paragraph: saying that bionix comes
> with stronger reproducibility guarantees than WMs based on general
> purpose languages is a claim that needs to be proven. I cannot see
> this from the current manuscript.
> * The sentence "By contrast, the WDL and NextFlow examples presented are
> valid workflow specifications without software definitions." standing
> alone without a clarification leaves the impression behind that
> Nextflow and WDL don't support defining the software environment. I
> know that they don't enforce it, but why not simply saying that, but
> also stating that they very well allow to. Also, I still think that
> software env definition has to be added to Example 10 for Nextflow and
> WDL. There is no point in leaving it out. You can simply mention that
> it is not enforced.
>
> # Minor comments
>
> * In the introduction, the distinction between envs and software
> versions seems artificially sharp. A package manager like conda
> handles both software, versions and dependencies as well as
> computational environment (conda envs). It is therefore a solution for
> both challenge 1 and 2. It is just a more lightweight way of
> environment management that containers or even VMs.
>
> Johannes Köster

Dear editor and reviewers,

Thank you for the additional feedback. We have addressed the final issues raised by the following:

1. Our discussion of other workflow managers has been expanded. We've added additional citations to workflow management software, as well as review papers and lists, under the "Existing workflow managers for computational biology" section.

2. To acknowledge in our comparisons that other systems are capable of using containers, we have added an example of this in WDL. We opted to add an additional WDL example, rather than modifying the existing one, for two reasons: 1) we feel that the existing, containerless example illustrates some important points, and 2) we wanted to stick with examples taken from other tools' documentation.

3. We have removed the claim that BioNix gives stronger reproducibility guarantees than alternatives based on a reproducibility stack. Though we believe that Nix does indeed provide such guarantees, our manuscript neither empirically demonstrates nor quantifies this.

4. Our introduction distinguishes between managing environments, managing software versions, and specifying workflows not because we believe that there are sharp distinctions between the three problems, but because historically they have been tackled separately. We have added an acknowledgement of existing efforts to tackle more than one problem at once, specifically mentioning Conda environments.

We hope you find the revisions satisfactory.

Kind regards,

The Authors

Close
