# Supplementary material for: Unifying package managers, workflow engines, and containers: Computational reproducibility with BioNix
Source: Gigascience. 2020 Nov 18;9(11):giaa121. doi: 10.1093/gigascience/giaa121 (PMC7672450; doi:10.1093/gigascience/giaa121)
Supplement: giaa121_Response_to_Reviewer_Comments_Revision_3 [file giaa121_response_to_reviewer_comments_revision_3.pdf]

Author's Response To Reviewer Comments

Close

Moved the GigaDB reference to the availability section and added license information as well.

Close
