# Supplementary material for: Unifying package managers, workflow engines, and containers: Computational reproducibility with BioNix
Source: Gigascience. 2020 Nov 18;9(11):giaa121. doi: 10.1093/gigascience/giaa121 (PMC7672450; doi:10.1093/gigascience/giaa121)
Supplement: giaa121_Reviewer_1_Report_Original_Submission — Konstantinos Krampis, PhD -- 10/7/2019 Reviewed [file giaa121_reviewer_1_report_original_submission.pdf]

Reviewer Report

Title: Unifying package managers, workflow engines, and containers: computational reproducibility with BioNix

Version: Original Submission **Date:** 10/7/2019

Reviewer name: Konstantinos Krampis, PhD

Reviewer Comments to Author:

Novel approach to composition of bioinformatics workflows, targeted to developers. Some minor additions to the paper are required:
1). HPC environment is mentioned, and the integration with queuing systems, how are jobs in the workflow that are dropped by the queuing system are handled in bionix ?
2). A little more comparison with existing workflow systems is needed in the discussion section (there is only a brief mention of Galaxy, for the bionix integration with it). How does for example bionix compare with nextflow, snakemake etc ? A comparison with points on features (present / absent in bionix) to the other workflow platforms would be useful in the discussion, for readers looking to compare and decide which platform to adopt.
3). Does bionix integrate with Docker and if not, any disadvantages from that ? I believe it should be considered as standard that modern bioinformatics workflow systems can run tools prepackaged in containers at any of the analysis steps included in the workflow (the use of containers is becoming pervasive in bioinformatics for multiple reasons). Look for example the nextflow platform, and also Galaxy which now enables tool execution from containers.

Level of Interest

Please indicate how interesting you found the manuscript: Choose an item.

Quality of Written English

Please indicate the quality of language in the manuscript: Choose an item.

Declaration of Competing Interests

Please complete a declaration of competing interests, considering the following questions:

- Have you in the past five years received reimbursements, fees, funding, or salary from an organisation that may in any way gain or lose financially from the publication of this manuscript, either now or in the future?
- Do you hold any stocks or shares in an organisation that may in any way gain or lose financially from the publication of this manuscript, either now or in the future?

- Do you hold or are you currently applying for any patents relating to the content of the manuscript?
- Have you received reimbursements, fees, funding, or salary from an organization that holds or has applied for patents relating to the content of the manuscript?
- Do you have any other financial competing interests?
- Do you have any non-financial competing interests in relation to this paper?

If you can answer no to all of the above, write 'I declare that I have no competing interests' below. If your reply is yes to any, please give details below.

No competing interests.

I agree to the open peer review policy of the journal. I understand that my name will be included on my report to the authors and, if the manuscript is accepted for publication, my named report including any attachments I upload will be posted on the website along with the authors' responses. I agree for my report to be made available under an Open Access Creative Commons CC-BY license (http://creativecommons.org/licenses/by/4.0/). I understand that any comments which I do not wish to be included in my named report can be included as confidential comments to the editors, which will not be published.

Choose an item.

To further support our reviewers, we have joined with Publons, where you can gain additional credit to further highlight your hard work (see: https://publons.com/journal/530/gigascience). On publication of this paper, your review will be automatically added to Publons, you can then choose whether or not to claim your Publons credit. I understand this statement.

Yes Choose an item.
