# Supplementary material for: Unifying package managers, workflow engines, and containers: Computational reproducibility with BioNix
Source: Gigascience. 2020 Nov 18;9(11):giaa121. doi: 10.1093/gigascience/giaa121 (PMC7672450; doi:10.1093/gigascience/giaa121)
Supplement: giaa121_Reviewer_2_Report_Original_Submission — Ola Spjuth -- 11/1/2019 Reviewed [file giaa121_reviewer_2_report_original_submission.pdf]

Reviewer Report

Title: Unifying package managers, workflow engines, and containers: computational reproducibility with BioNix

Version: Original Submission **Date:** 11/1/2019

Reviewer name: Ola Spjuth

Reviewer Comments to Author:

The author describes BioNix, submitted as a technical note. The paper is well written, motivates the development well, and discusses relevant previous work. Also, we were able to build and run some simple examples locally on a Linux laptop. Despite being a technical note, my main concern is that we believe that the case studies presented in Life Science are very thin.

== Major concerns

Main The authors should present much more in-depth analysis of concrete applications in life science. Now the paper is almost exclusively a technical description, and the
section "Real world use of BioNix" is very short and lacks details. No code is presented. If more and longer analyses on life science data are available then it could be accepted as a Technical Note in Gigascience. Else, I would consider this manuscript to be more suited to a more specialized technical journal. Further, with such a high focus on reproducibility, make sure the readers can reproduce all the cases in the "Real world use" section.

Apart from the main concern, also the following points need to be addressed:

1. There is no section discussing known limitations. All design desicions come with certain tradeoffs. Thus for clarity and fairness to the reader, we think such a section should be included.
2. There is one area that could use some more elaboration: The handling of multiple inputs and outputs. This is a common case in many types of workflows, and in some cases two outputs from one process need to take different downstream routes, such as when splitting a dataset into a test- and a training set for machine learning.
3. The examples lack an overview describing what happens in each example. This makes it a bit hard to know what to expect from the examples.
4. An area that can often get cumbersome in some cases is where only the dependencies between stages or processes are defined, but not how each individual output are connected to downstream processes. A discussion of how this issue is handled in BioNix would be very helpful.
5. Consider citing the following highly relevant papers:

- https://www.nature.com/articles/s41592-018-0046-7
- https://academic.oup.com/gigascience/article/8/5/giz044/5480570

== Minor concerns

Page 1, column 2, 5th row from bottom:
"the one" perhaps should be "one"?
Page 2, column 1, you say:

"inflexible verbosity of static conguration files"
- Is static configuration files really known for verbosity? Inflexibility, yes, but verbosity?
Page 3, column 1, you say:
"Alternatively, one can mimic multi-argument functions using higher-order functions - i.e., functions which return functions."
- This is not very clear how that works, and an example or more elaborate explanation would help.
Page 3, column 2: Broken sentence?
"For most of our stages we do not pass in any additional options,
and so the first However"
Page 4, column 2: This sentence needs a little more explanation:
" However, as submission is a (relatively benign) side effect, builds cannot be realised using sandboxing."
Page 5, column 2:
"written directly"
Should probably be:
"written by humans"
Page 5, column 2, under "Conclusions"
"modifying" seems it should be "modify"

Level of Interest

Please indicate how interesting you found the manuscript: Choose an item.

Quality of Written English

Please indicate the quality of language in the manuscript: Choose an item.

Declaration of Competing Interests

Please complete a declaration of competing interests, considering the following questions:

- Have you in the past five years received reimbursements, fees, funding, or salary from an organisation that may in any way gain or lose financially from the publication of this manuscript, either now or in the future?
- Do you hold any stocks or shares in an organisation that may in any way gain or lose financially from the publication of this manuscript, either now or in the future?
- Do you hold or are you currently applying for any patents relating to the content of the manuscript?
- Have you received reimbursements, fees, funding, or salary from an organization that holds or has applied for patents relating to the content of the manuscript?
- Do you have any other financial competing interests?
- Do you have any non-financial competing interests in relation to this paper?

If you can answer no to all of the above, write 'I declare that I have no competing interests' below. If your reply is yes to any, please give details below.

I declare that I have no competing interests

I agree to the open peer review policy of the journal. I understand that my name will be included on my report to the authors and, if the manuscript is accepted for publication, my named report including any attachments I upload will be posted on the website along with the authors' responses. I agree for my report to be made available under an Open Access Creative Commons CC-BY license (http://creativecommons.org/licenses/by/4.0/). I understand that any comments which I do not wish to be included in my named report can be included as confidential comments to the editors, which will not be published.

Choose an item.

To further support our reviewers, we have joined with Publons, where you can gain additional credit to further highlight your hard work (see: https://publons.com/journal/530/gigascience). On publication of this paper, your review will be automatically added to Publons, you can then choose whether or not to claim your Publons credit. I understand this statement.

Yes Choose an item.
