# Supplementary material for: Unifying package managers, workflow engines, and containers: Computational reproducibility with BioNix
Source: Gigascience. 2020 Nov 18;9(11):giaa121. doi: 10.1093/gigascience/giaa121 (PMC7672450; doi:10.1093/gigascience/giaa121)
Supplement: giaa121_Reviewer_3_Report_Original_Submission — Johannes KÃ¶ster -- 11/4/2019 Reviewed [file giaa121_reviewer_3_report_original_submission.pdf]

Reviewer Report

Title: Unifying package managers, workflow engines, and containers: computational reproducibility with BioNix

Version: Original Submission **Date:** 11/4/2019

Reviewer name: Johannes KÃ¶ster

Reviewer Comments to Author:

The manuscript describes BioNix, a unified workflow management system that uses the nix framework to integrate package management with workflow management.
The central innovation of BioNix that it finds a way to express both the workflow steps as well as software deployment steps via the same, functional, Nix DSL.
The paper is written in an understandable and well structured way. The central innovation mentioned above is an interesting addition to what is available in the field, and indeed deserves to be published. Unfortunately, the paper in its current form, in particular when comparing with other approaches is sometimes unnecessarily biased and not properly justified. It seems like the manuscript tries to convince the reader that the presented approach is superior to others. However, this claim is never more than a plain hypothesis, as any given arguments are not convincing. Instead, I suggest to simply present the new approach and maybe briefly compare to others without making a subjective judgement.
Please find details below.
Major comments
* The introduction correctly lists three pillars of in silico reproducibility. However, there is more to it. First, scalability is an issue as well. People need to be able to scale a workflow to their computational environment (e.g. cluster or cloud) ideally without the need to modify the workflow definition itself. Second, transparency and readability is important as well. Apart from computational reproduction as a black box, it is important that people can judge over the taken steps in terms of parameters and code. Hence, it is important that a workflow is in large parts human readable without knowing the used definition language.
* Page 2: sandboxing is indeed a nice feature, which, as the authors correctly conclude, can also be achieved with containers. The authors should clarify whether it has to be enabled/disabled globally or if it is possible to change this per step. Also, they should highlight more prominently that BioNix only supports it for local (or cloud?) execution, not for cluster execution.
* Page 2: it is first mentioned that the nixpkgs import fixes the versions of all used software, and later the authors mention that software versions can be defined per stage. The authors should provide an example of the latter.
* Page 2, paragraph 3: the statement "inflexible verbosity of configuration files ... error-prone power of general purpose language" is fairly judgemental, without providing any reasoning. If the authors make such a statement, they need to provide particular reasons in detail, and also highlight the upsides of both. Also, an aspect not considered here is that there are situations where config files are more suitable than a functional language and as well where a general purpose programming language is more

suitable than a functional specification language like Nix. One could argue that this is the case for workflow management. It combines the need for declaring options, parameters, software dependencies (which configuration file languages are designed for), with the need for declaring steps (obviously a declarative task) and the need for flexibility in certain corner cases, e.g. complex aggregations, conditional execution (for which the full power of general purpose programming languages might be needed). Hence, there is certainly a case for not unifying all the different aspects of workflow definition in a single language. I would like to see the paper discussing this openly, without making a judgement. An example for such a system is Snakemake, which provides a declarative syntax for workflow step definition on top of Python, a general purpose language, in combination with YAML for configuration files and conda software environment definition files for ensuring controlled execution environments. Since Snakemake is among the most widely used workflow managers it should definitely be discussed along with Galaxy, Nextflow, WDL and CWL here.
* If understood correctly, the submission of a BioNix workflow to a cluster system needs the modification of the workflow definition by adding submission functions. This would limit reproducibility (and should be communicated as such), because the workflow potentially needs to be adapted before execution on a new system.
* The comparison with other systems is extremely biased. See details below.
* For example, both Snakemake and Nextflow support streaming between jobs and conditional execution, which does not seem to be supported by BioNix. It is of course infeasible to list the union of all features of all workflow management systems, but (a) this should be clearly communicated and (b) one should try to find a balance of advantages and disadvantages of all systems, or even better just list available and missing features and leave the judgement to the reader.
* The authors mention that the nextflow definition is more implicit than BioNix, and this may increase difficulty of interpretation. First, from the example, I cannot see this increased implicitness. Second, implicitness can also be an advantage, and explicitness also has downsides. Please communicate this openly.
* I could not follow the comparison with CWL. First, I do not agree that CWL is low-level. I would agree with a statement saying that CWL is quite verbose and hard to read. However, at least for somebody not familiar with Nix, this is also the case for BioNix. Readability for non-authors is a very important aspect, and both systems are not the best I have seen so far in this regard.
* The statement that Nix integration in Galaxy would lead to a better control of the computational environment has to be justified. What makes Nix better than Conda or Containers, in terms of control?
* In the comparison with Guix, the manuscript mixes a statement that I would consider neutral (functional vs. data structures) with something negative (no types). Maybe try to separate the two, such that functional vs DS does not inherit a negative connotation.
* The conclusion, well, draws the conclusion that BioNix workflows are easier to specify, share execute, modify and reproduce. This is a very general statement that I do not believe given the arguments in the manuscript before. Why is the functional language of Nix easier to modify than e.g. a Nextflow or WDL-based workflow definition? In particular why is it an advantage to integrate everything into one language? For example, speaking about modification: if one just wants to modify a software version or a configuration parameter, all one has to do with a Snakemake workflow is to edit two YAML files (the respective Conda software environment, and the config file). This seems easier than with BioNix at first

sight.
* Example 5 compares BioNix with Nextflow and WDL. The caption says that the Nextflow pipeline does not capture the software used. I am sorry to say this, but such a statement almost upsets me. Nextflow is very well able to define this. By adding a single line to each step, one could either define a container or an isolated conda environment. I suggest to carefully read the documentation of tools before writing such comparisons.
# Minor comments
* Various paragraphs on page 3 are somehow messed up (incomplete sentences). Please carefully check them.
* Page 4, right column, first paragraph: scheduler, not schedulerS.

Level of Interest

Please indicate how interesting you found the manuscript: Choose an item.

Quality of Written English

Please indicate the quality of language in the manuscript: Choose an item.

Declaration of Competing Interests

Please complete a declaration of competing interests, considering the following questions:

- Have you in the past five years received reimbursements, fees, funding, or salary from an organisation that may in any way gain or lose financially from the publication of this manuscript, either now or in the future?
- Do you hold any stocks or shares in an organisation that may in any way gain or lose financially from the publication of this manuscript, either now or in the future?
- Do you hold or are you currently applying for any patents relating to the content of the manuscript?
- Have you received reimbursements, fees, funding, or salary from an organization that holds or has applied for patents relating to the content of the manuscript?
- Do you have any other financial competing interests?
- Do you have any non-financial competing interests in relation to this paper?

If you can answer no to all of the above, write 'I declare that I have no competing interests' below. If your reply is yes to any, please give details below.

I declare that I have no competing interests

I agree to the open peer review policy of the journal. I understand that my name will be included on my report to the authors and, if the manuscript is accepted for publication, my named report including any attachments I upload will be posted on the website along with the authors' responses. I agree for my report to be made available under an Open Access Creative Commons CC-BY license (http://creativecommons.org/licenses/by/4.0/). I understand that any comments which I do not wish to be included in my named report can be included as confidential comments to the editors, which will not be published.

Choose an item.

To further support our reviewers, we have joined with Publons, where you can gain additional credit to further highlight your hard work (see: https://publons.com/journal/530/gigascience). On publication of this paper, your review will be automatically added to Publons, you can then choose whether or not to claim your Publons credit. I understand this statement.

Yes Choose an item.
