# Supplementary material for: Unifying package managers, workflow engines, and containers: Computational reproducibility with BioNix
Source: Gigascience. 2020 Nov 18;9(11):giaa121. doi: 10.1093/gigascience/giaa121 (PMC7672450; doi:10.1093/gigascience/giaa121)
Supplement: giaa121_Reviewer_3_Report_Revision_1 — Johannes KÃ¶ster -- 5/7/2020 Reviewed [file giaa121_reviewer_3_report_revision_1.pdf]

Reviewer Report

Title: Unifying package managers, workflow engines, and containers: computational reproducibility with BioNix

Version: Revision 1 **Date:** 5/7/2020

Reviewer name: Johannes KÃ¶ster

Reviewer Comments to Author:

First, I'd like to thank the authors for carefully considering my previous comments. Some issues remain, which I'd like to address below.
Major comments
* The comparison with other systems is still quite short. Nevertheless it is necessary (also in the interest of the authors) to show that they know the field before making claims and developing something new. Of course I completely understand the reasoning behind not making feature matrices and full comparisons, this is the job of review papers. Still, it is good scientific practice to briefly summarize the _entire_ field when publishing something new. At least, all major workflow management systems should be named and cited (check out this for a list: https://github.com/pditommaso/awesome-pipeline). The authors could then say that you chose some representatives for comparison... The chosen set the authors have now is already quite good, because it represents the different niches.
* Page 2, left part, second-last paragraph: saying that bionix comes with stronger reproducibility guarantees than WMs based on general purpose languages is a claim that needs to be proven. I cannot see this from the current manuscript.
* The sentence "By contrast, the WDL and NextFlow examples presented are valid workflow specifications without software definitions." standing alone without a clarification leaves the impression behind that Nextflow and WDL don't support defining the software environment. I know that they don't enforce it, but why not simply saying that, but also stating that they very well allow to. Also, I still think that software env definition has to be added to Example 10 for Nextflow and WDL. There is no point in leaving it out. You can simply mention that it is not enforced.
# Minor comments
* In the introduction, the distinction between envs and software versions seems artificially sharp. A package manager like conda handles both software, versions and dependencies as well as computational environment (conda envs). It is therefore a solution for both challenge 1 and 2. It is just a more lightweight way of environment management that containers or even VMs.
Johannes KÃ¶ster

Level of Interest

Please indicate how interesting you found the manuscript: Choose an item.

Quality of Written English

Please indicate the quality of language in the manuscript: Choose an item.

Declaration of Competing Interests

Please complete a declaration of competing interests, considering the following questions:

- Have you in the past five years received reimbursements, fees, funding, or salary from an organisation that may in any way gain or lose financially from the publication of this manuscript, either now or in the future?
- Do you hold any stocks or shares in an organisation that may in any way gain or lose financially from the publication of this manuscript, either now or in the future?
- Do you hold or are you currently applying for any patents relating to the content of the manuscript?
- Have you received reimbursements, fees, funding, or salary from an organization that holds or has applied for patents relating to the content of the manuscript?
- Do you have any other financial competing interests?
- Do you have any non-financial competing interests in relation to this paper?

If you can answer no to all of the above, write 'I declare that I have no competing interests' below. If your reply is yes to any, please give details below.

I declare that I have no competing interests

I agree to the open peer review policy of the journal. I understand that my name will be included on my report to the authors and, if the manuscript is accepted for publication, my named report including any attachments I upload will be posted on the website along with the authors' responses. I agree for my report to be made available under an Open Access Creative Commons CC-BY license (http://creativecommons.org/licenses/by/4.0/). I understand that any comments which I do not wish to be included in my named report can be included as confidential comments to the editors, which will not be published.

Choose an item.

To further support our reviewers, we have joined with Publons, where you can gain additional credit to further highlight your hard work (see: https://publons.com/journal/530/gigascience). On publication of this paper, your review will be automatically added to Publons, you can then choose whether or not to claim your Publons credit. I understand this statement.

Yes Choose an item.
